# Micronutrient bioavailability: concepts, influencing factors, and strategies for improvement

**DOI:** 10.3389/fnut.2025.1646750

**Published:** 2025-11-19

**Authors:** James D. Richards, Héctor Cori, Maike Rahn, Kristen Finn, Juan Bárcena, Alexandros K. Kanellopoulos, Szabolcs Péter, Anneleen Spooren

**Affiliations:** 1 Health, Nutrition & Care, dsm-firmenich, Plainsboro, NJ, United States; 2 Health, Nutrition & Care, dsm-firmenich, Santiago, Chile; 3 Health, Nutrition & Care, dsm-firmenich, Kaiseraugst, Switzerland

**Keywords:** micronutrient, bioavailability, absorption, vitamin, mineral, supplementation, fortification

## Abstract

The human diet provides a range of macronutrients and micronutrients, which are metabolized for energy and used to support all aspects of life. The extent to which these nutrients are absorbed in a form that can be used by metabolic processes, or stored for later use, is termed bioavailability. Certain dietary factors increase the bioavailability of micronutrients: bioavailability can be enhanced by different vitamin forms (e.g., calcifediol is more bioavailable than cholecalciferol; methylfolate is more bioavailable than folic acid), and by the food matrix and nutrient interactions (e.g., fat increases absorption of fat-soluble vitamins; multiple vitamins support iron absorption and metabolism). Conversely, plant-based foods exhibit reduced micronutrient bioavailability, due to entrapment in cellular structures and binding by antagonists such as phytate and fiber. Host factors also impact micronutrient availability. A healthy gastrointestinal microbiota can increase the absorption of vitamins and minerals, and certain life stages (e.g., pregnancy and lactation) are characterized by increased absorptive capacity. In contrast, the elderly exhibit reduced ability to absorb certain vitamins, and bacterial overgrowth/dysbiosis can reduce the availability of several vitamins. Several medications reduce vitamin absorption and status. Reduced bioavailability, poor quality diets, lower micronutrient content in foods due to soil depletion and climate change, and decreasing micronutrient intakes lead to widespread, global deficiencies. This in turn contributes to higher prevalence of non-communicable diseases such as anemia, osteoporosis, cardiovascular disease, and blindness; decreased growth; impaired immunity and increased incidence of infections; and increased mortality rates. Strategies to improve bioavailability and nutrient status are crucial and align with United Nations Strategic Development Goals 2 and 3. Vitamins and minerals added to foods or taken as supplements generally are at least as bioavailable as those endogenously in foods, and often more so. In addition, certain technologies are available to further increase micronutrient bioavailability. These include permeation enhancers, lipid-based formulations, nutrient compounding/encapsulation, and phytase to increase bioavailability of minerals trapped by phytic acid. Solutions such as these will help supply highly absorbable and utilizable vitamins and minerals, help close widespread nutritional gaps, and support adequate nutritional status and optimal health across diverse populations worldwide.

## 1 Introduction

The foods people consume provide the nutrients required for all aspects of life, including growth and development, reproduction, and maintaining health (1). Nutrients provide the nourishment required to maintain the functioning of organs and organ systems including the brain and nervous system, the immune system, the gastrointestinal system, bone, muscle, skin, and many others. Proper nutrition also helps reduce the risk of a wide variety of communicable (infectious) illnesses and noncommunicable diseases (NCDs). Furthermore, an individual’s nutrient intakes and status play important roles supporting healthy aging processes (2, 3). Therefore the diets we consume, and the nutrients those diets provide, are critical to human health (1).

Ideally, the regular, optimal intake of all these nutrients at required levels (e.g., the RDA), and the adequate status of these nutrients, would be achieved through the consumption of a diverse and well-balanced diet. However, recent publications confirm that even in medium and high-income regions, where there is access to diverse and well-balanced diets, micronutrient deficiencies remain common (4, 5). Indeed, micronutrient intakes and status have long been inadequate, and the consumption of certain micronutrients continues to decrease in many areas around the world, including in high income countries. As one example, vitamin C intake has decreased substantially in recent years in Korea, China, and the United States (6–8). These insufficient intakes, even in the presence of access to healthy diets, contribute to the lingering and wide prevalence of nutrient deficiencies globally (see section 4.3 Decreasing Rates of Micronutrient Intake below).

Furthermore, the intrinsic content of nutrients in foods does not guarantee their utility in the body. First, not all nutrients in food are stable across the shelf life and during food preparation (e.g., heating, freezing or other processes commonly applied in processed foods). Second, only a fraction of nutrients in foods are absorbed, transported, and delivered to the cells or stored in a form that can be utilized to support metabolic processes. This proportion of nutrients are those that are considered to be “bioavailable” (9, 10). Bioavailability can vary widely depending on diet-related factors including the nutrient and its form, the food matrix, and the existence of dietary antagonists limiting the absorption of the nutrient. Certain factors including the individual’s age, physiological state, genetic variability, gender, nutrient status, and the existence of different disease states can impact nutrient bioavailability (11).

The challenges in consuming a well-balanced diet, low micronutrient bioavailability because of the above-mentioned factors, and the variability in nutrient levels from different foods (e.g., due to climate change and seasonal variations), result in nutrient inadequacies and deficiencies. Indeed, recent research indicates that in high income countries, approximately half of non-pregnant women between 15 and 49 years of age are deficient in at least one of iron, zinc, and folate. Globally, that percentage is approximately 69%, representing 1.2 billion women (12). In 2014 it was reported that between 37 and 88% of the global population has inadequate vitamin D status, depending on the chosen threshold of adequacy (<50 nmol/L or <75 nmol/L circulating 25(OH)D, respectively) (13). A new meta-analysis of over 600 studies since 2011 confirms that almost half of the global population still has circulating 25(OH)D levels below 50 nmol/L (5). Furthermore, a recent study estimates that at least 5 billion people worldwide have inadequate intakes of iodine, vitamin E, or calcium from food, excluding the intake from fortification and supplementation. Approximately 4.9 billion do not consume enough iron, and more than 4 billion do not take in enough riboflavin, folate, or vitamin C (4).

As global fortification or supplementation of most of these nutrients is uncommon (iodine, riboflavin, and folic acid being notable exceptions), these data show that the majority of the world population experiences inadequate dietary micronutrient intake (4). This situation leads to a broad spectrum of negative health impacts including decreased growth, compromised immune function and consequent increased incidence of infectious disease, and increased prevalence of NCDs such as osteoporosis, cardiovascular disease, anemia, blindness, and cancer (14–17). The World Health Organization (WHO) estimates that 40% of children aged 6–59 months, 37% of pregnant women, and 30% of women aged 15–49 years are anemic, for example (14). As such, micronutrient deficiencies can have profound impacts on health, as well as quality and duration of life (18). There is also increasing emerging evidence that nutrient depletion or insufficient nutrient supply plays a key role in the aging process and age-related NCD development (19). Supplementation and food fortification are safe, effective, and low-cost ways to provide highly bioavailable nutrients to help eliminate nutritional gaps and thus improve the overall health of individuals and the population. As one example, correcting vitamin D deficiency only costs between 0.06–0.2% of the expenses associated with assessing and treating the comorbidities associated with it. Indeed, vitamin D supplementation is estimated to cost approximately $12 U.S. per person, whereas the cost of treating the complications related to vitamin D deficiency, including early death, ranges from $6,000 U.S. to $18,000 U.S. annually per affected individual (20).

Within this context, this review will focus on different aspects of nutrient bioavailability, including its definition and how it is measured, factors that influence bioavailability, and why is it important for human nutrition and health. We will also provide case studies that illustrate strategies to enhance micronutrient bioavailability.

## 2 Bioavailability: what is it and how is it measured?

Despite its importance, there is no clear consensus on how to define bioavailability (10, 21). Recently, the European Food Safety Authority (EFSA) has described bioavailability in conceptual terms, as the “availability of a nutrient to be used by the body” (9). Several authors describe bioavailability as the fraction of an ingested nutrient that enters the bloodstream or becomes available for use in normal physiological functions and storage in the body (11, 22–24). The U.S. Institute of Medicine (IOM) (now National Academy of Medicine [NAM]) states “the bioavailability of a nutrient can be defined as its accessibility to normal metabolic and physiologic processes” (25). More detailed, mechanistic definitions include “the proportion of an ingested nutrient that is released during digestion, absorbed via the gastrointestinal tract, transported and distributed to target cells and tissues, in a form that is available for utilization in metabolic functions or for storage” (10). Common to these definitions is the concept that to be bioavailable, a nutrient (including a micronutrient) in a food (or supplement) must be absorbable and transported in a form that can be used in metabolism or stored by the body for future utilization.

Methods to measure bioavailability are varied and depend at least partly on the nutrient being investigated. A variety of experimental approaches have been used, including the utilization of chemical data (e.g., dissociation tests), *in vitro* studies simulating human digestion (e.g., to provide evidence to what extent and with what kinetics a nutrient can be released from a source and enter cells that are intended to mimic the intestinal epithelium), animal models (e.g., tissue uptake and other biomarkers of absorption), and human studies (9). Interpretation of assays can be complicated by the metabolic processes & transformations to which nutrients can be subjected. The nutrient form that is supplied is not always equal to the nutrient that is transported or the one that is stored, and some of these nutrient forms can be short-lived and therefore difficult to detect. In some cases, there is no universal consensus on the most relevant biomarker to be measured and in which biosample. Assessing which nutrient form to measure is an important step for any bioavailability assay. Studies in relevant human populations are considered most informative and can yield results that differ from those in animal models. As one example, studies in rodent models generally found differences in bioavailability of synthetic vs. natural vitamin C. In contrast, there were no differences between sources in humans (26).

One of the most common methods for measuring the bioavailability is the balance study, which measures the difference between ingestion of a nutrient and its excretion (10, 27, 28). “Ileal digestibility” measures the difference between the ingested amount and that remaining in ileal contents and is considered a reliable indicator for apparent absorption (10, 29, 30). Another variation measures fecal content of the nutrient in question rather than ileal content, assuming that undigested nutrients will be excreted. However, it should be noted that certain species of colonic microbiota can degrade or synthesize certain vitamins, for example B vitamins, which could alter results from this approach when the bioavailability of these vitamins are being assessed (10, 29, 30). Furthermore, looking at the difference between intake and excretion does not provide any insights into the availability of the nutrient in question to specific cells, tissues, or organs in the body, and thus to the potential utilization of the nutrient for metabolic processes. In other studies, stable or radioactive isotopes of nutrients are utilized to monitor the nutrient’s absorption and retention by the body (10, 27). Labeled isotopes can be used as well when validated biomarkers for the status of a particular nutrient exist, for example the incorporation of labeled iron into red blood cells. Other methods include accumulation of the nutrient in blood, tissues, the whole body, or urine (10, 27, 31). In some cases, a metabolite of the ingested nutrient is the accepted biomarker. Perhaps the best example here is with vitamin D, where circulating 25(OH)D is universally considered the most appropriate biomarker for vitamin D status (32).

A distinction can also be made between the measurement of “true” bioavailability and relative bioavailability. True bioavailability of a nutrient can be estimated in balance studies by correcting for endogenous losses of the nutrient in the intestinal tract, or by comparing the response to an oral dose of the nutrient in comparison to a dose delivered intravenously (10, 28–30). In practice, however, relative bioavailability typically is measured. Relative bioavailability studies estimate the efficiency of uptake of a nutrient in one given source or form (for example, zinc in zinc oxide), in relation to that of a reference source or form (e.g., zinc in zinc sulfate), under identical experimental conditions. In animal models, the nutrient of interest is typically measured in a tissue considered to be a reliable indicator for that nutrient—for example, zinc content is often measured in bone—or in the entire animal. In this case, one would conclude the bioavailability of the test form as a proportion or percentage of the bioavailability of the reference from (9, 10, 30, 33). Again however, there is still the limitation that these studies help estimate the uptake of a nutrient, but often provide little information regarding the distribution of the nutrient to cells and tissues of interest.

Thus, there are many experimental approaches to measuring bioavailability, that vary both in their experimental complexity and their precision in providing a bioavailability estimate (e.g., does the chosen method correct for endogenous losses?; does it utilize the most appropriate biomarker?). Thus, care must be taken to employ the best approach to address the specific questions that are asked (e.g., is absolute or relative bioavailability being sought?), and when interpreting the resulting data or comparing data across various studies.

## 3 Factors that impact bioavailability

**Figure 1:**
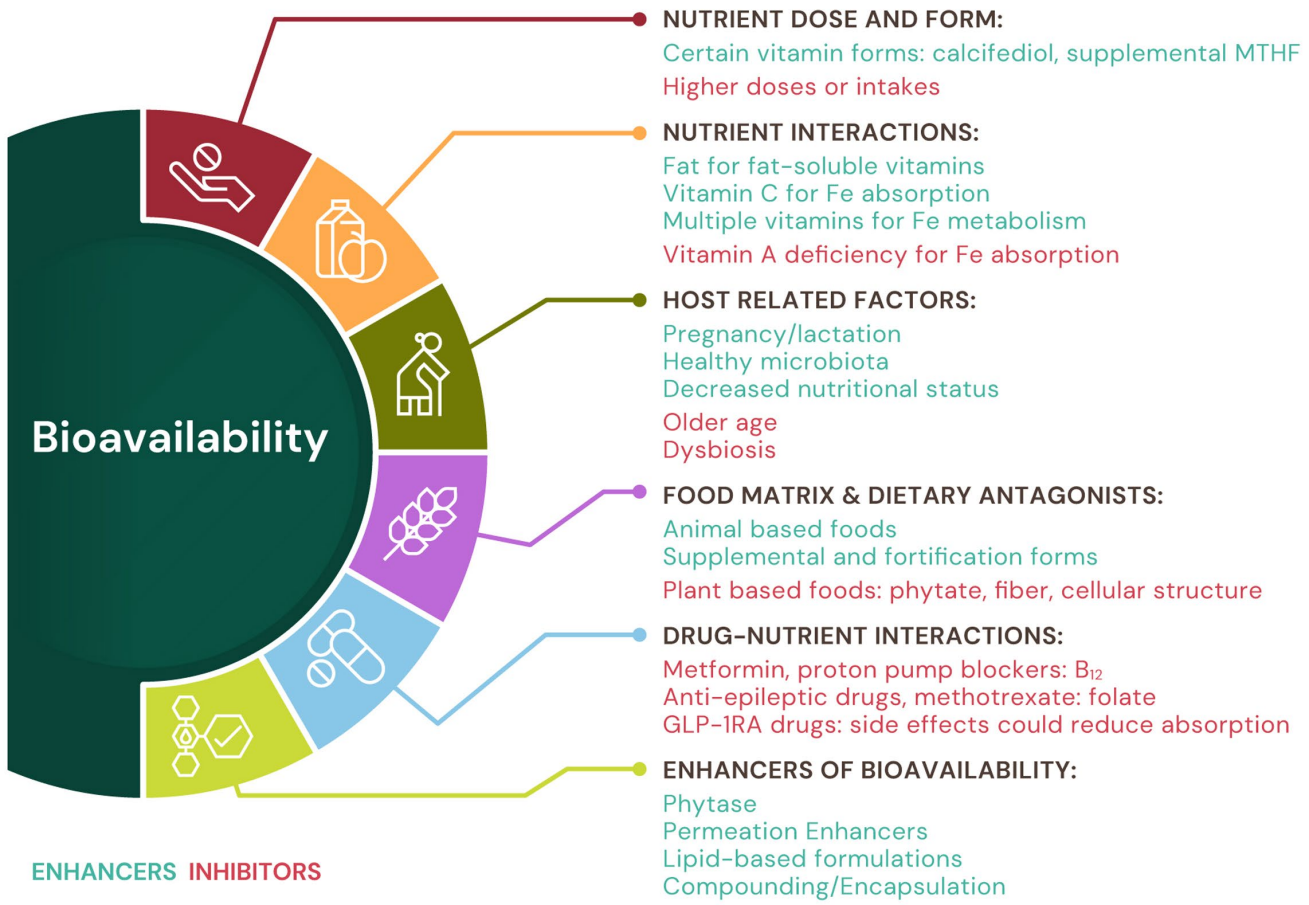
Factors that impact bioavailability of micronutrients. The bioavailability of a given micronutrient can be impacted by a wide variety of enhancing and inhibiting factors, including those that are diet-related and those that are host-related. Technologies to enhance micronutrient bioavailability also exist. See text for details. MTHF, methylfolate.

The bioavailability of a given micronutrient can be impacted by a wide variety of factors, including those that are diet-related and those that are host-related (Figure 1) (9, 11, 22, 34). Dietary factors that affect bioavailability are numerous and include the type of micronutrient (fat or water soluble vitamin, or mineral), form and dose of the nutrient, the dietary matrix (e.g., plant or animal based), interactions between the nutrient in question and other components of the diet that can inhibit or enhance absorption (e.g., fat enhances the absorption of fat soluble vitamins), and the impact of food processing and preparation (e.g., cooked vs. raw). Host-related factors include age (e.g., infants vs. adults vs. elderly), physiological state (e.g., pregnancy, menstruation, or menopause), metabolic pathways & nutrient transporter activity (e.g., feedback loops triggered by nutrient presence or nutrient transporter desensitization, such as the hepcidin-iron loop), nutrient status of the host (e.g., deficient vs. adequate status), health status of the host (e.g., healthy vs. certain disease states, including obesity vs. healthy BMI, and nutrient-drug interactions), and intestinal & organ factors (e.g., microbiota, reductions in gastric acid secretion, reductions in liver function for example in the case of vitamin D) (11). While it is beyond the scope of this paper to consider all the factors listed above, we will discuss a few important examples below, that are also summarized in Supplementary Table 1 (dietary factors) and Table 1 (host factors).

**Table 1:** Host factors that can impact bioavailability.

Factor	Population, or nutrient status	Impact	Mechanism	References
Prolonged low nutrient intake	All	Increased bioavailability	Increased absorptive capacity and renal nutrient retention	(11)
Life Stage	Elderly	Reductions in bioavailability of B vitamins, vitamin D, and calcium	Changes in secretory and absorptive capacity; Decreased intrinsic factor, stomach acid, and proteases; Vitamin D affects Ca absorption	(11, 61, 65–67)
	Pregnancy & lactation	Increased absorption and/or renal retention of Ca, Zn, Fe, Cu	Effects at least partially due to estrogen	(11, 70–72)
	Menopause	Decreased absorption of Ca	Decreased estradiol; Estrogen therapy improves absorption	(73–76)
Disease/medication	Diabetics taking Metformin	Decreased status of B_12_, and potentially other B vitamins, vitamin D, and Mg	Reduces absorption/status. B_12_ supplementation effective at preventing or treating deficiency, and potentially alleviating neuropathy	(81–83)
	Cancer	Malnutrition in 40–80% of patients	Systemic inflammation; anorexia; early satiety, malabsorption, nausea and vomiting, drug side effects, etc.; ESPEN provides strong recommendation that vitamins and minerals be supplied at the RDA	(86–88)
	Proton pump inhibitors/reduced stomach acid	B_12_ status	Reduction of food, but not supplemental, B_12_ absorption	(89)
	Statins	Reduce vitamin D synthesis	Vitamin D synthesized from cholesterol	(92)
	Those taking medications that reduce fat absorption	Reduced vitamin A, D, E, and K uptake	Decreased absorption	(93)
	Intestinal infectious diseases & diarrhea	Decreased uptake of multiple micronutrients	Reduced absorption	(18)
	Digestive impairments (bile acids, pancreatic insufficiency)	Vitamin D	Malabsorption: Subjects with impaired bile acid release or pancreatic insufficiency both demonstrated significantly reduced absorption of vitamin D, but not calcifediol	(32, 154)
	Anticoagulants (for thrombotic disorders)	Vitamin K	Acquired deficiency: Acquired cellular vitamin K deficiency and a decrease in the synthesis of the vitamin K-dependent plasma clotting factors	(46)
	Obesity	Decreased status reported for vitamin D (but lesser impact with calcifediol), iron, vitamins B_1_, B_6_, B_12_, folate, vitamin A, vitamin C, magnesium, selenium, zinc and potassium.	Increased body volume; partitioning of fat-soluble vitamins to fat stores; metabolic and microbiota differences; inflammation; poor diets.	(141, 142, 144, 149–151)
	Alcohol abuse	Thiamine deficiency	Impaired absorption, poor diet, impaired liver storage	(205)
Nutrient status	All	Iron status	Iron absorption is downregulated with high status	(94)
	Vitamin A deficiency	Iron absorption & mobilization	Impaired with vitamin A deficiency, and enhanced with vitamin A supplementation	(98)
	Vitamin C status	Iron absorption & mobilization	Enhanced with vitamin C	(98)
	Riboflavin deficiency	Hematological response to iron	Enhanced with riboflavin; riboflavin deficiency increases anemia	(98)
	Vitamin D status	Increased serum iron	Vitamin D promotes export of iron from tissues	(101)
Microbiota	All	Two-way modulation of vitamin & mineral status	The gut microbiota can variously influence the bioavailability of micronutrients, as well as be influenced by micronutrient supplementation, with implications for health	(103, 104)
	Dysbiosis (SIBO)	Decreased micronutrient bioavailability	SIBO associated with villous atrophy and inflammation, decreased absorptive capacity, deconjugation of bile acids, malabsorption	(107, 108)

### 3.1 Dietary factors

Several dietary factors can impact the bioavailability of micronutrients, including the type of micronutrient (fat soluble vitamins, water soluble vitamins, minerals), the dietary matrix, the vitamin form, and dose and stability. This section will address these factors.

#### 3.1.1 General considerations: type of micronutrient

Micronutrients encompass both vitamins and minerals, with vitamins being subdivided into both water-soluble and fat-soluble categories. The water-soluble vitamins include the B vitamins and vitamin C, and the fat-solubles include vitamins A, D, E, and K (1). The general properties of the different vitamin classes can impact the factors that modify their bioavailability. Fat soluble vitamin uptake, whether endogenous or supplemental, involves bile-acid mediated emulsification and formation into micelles for absorption. Adequate dietary fat improves the absorption of these vitamins, whereas any condition that decreases bile production can inhibit uptake (11). When compounding all these vitamins into enhanced vitamin forms for fortification/supplementation, the emulsion droplet size impacts bioavailability, where optimal absorption is achieved with sizes below 1 micron (see Section 7.4). This contrasts with water-soluble vitamins, which are absorbed directly from the intestines without being impacted by these same processes (1, 11). This is not to say that there aren’t other factors that impact water soluble vitamins, and many are discussed below, including binding by components of the plant matrix. In many instances, though, factors that inhibit uptake of water-soluble vitamins are more impactful on endogenous vitamins than supplemental vitamins (10, 21). Minerals are quite different in that they are inorganic ions often found as salts, or in complexes with amino acids, organic acids, or peptides (1). They tend to have relatively low bioavailability, and are highly susceptible to binding by antagonists such as phytate, polyphenols, fiber, and other constituents in plants which inhibit binding to their receptors and thus their uptake (10, 11). Supplemental forms of minerals can vary greatly in their bioavailability and reactivity with the food matrix. For example ferrous iron is more bioavailable yet more reactive than ferric iron, zinc sulfate is more bioavailable in general than zinc oxide, and complexed mineral forms tend to be more bioavailable than salt forms (18, 33, 35–38).

#### 3.1.2 Dietary matrix

The food matrix can greatly impact bioavailability. A recent review on the bioavailability of 13 vitamins and choline concluded that in general, vitamins in foods from animal sources are more bioavailable than vitamins from plant sources (10). There are a variety of reasons for this. First, certain vitamins can be trapped in or bound to the food matrix in plants. Niacin for example can be linked to carbohydrates or peptides in grains and not fully released, in which case its bioavailability will be low unless the grains are subjected to alkaline treatment, as occurs with the making of tortillas (39, 40). Historically, poor bioavailability of the nicotinic acid form of niacin in corn was an important cause of pellagra (1). Similarly, entrapment in the insoluble cellular structure of certain plants has also been described to reduce the bioavailability of provitamin A carotenoids (41) and food folates and other B vitamins (11, 40, 42).

Second, plants contain several dietary antagonists that can reduce the absorbability of certain micronutrients. The classical example here is the presence of phytic acid, which is the primary storage form of phosphorus in legumes, cereal grains, and certain seeds. Phytic acid chelates metal ions, particularly calcium, zinc, and iron, and dramatically reduces their intestinal uptake (43–45). Other metal ion inhibitors include polyphenols and fibers (10, 11, 45). Indeed, plant-based iron is less bioavailable than heme-bound iron; the US IOM (now NAM) reports that iron is approximately 44% less bioavailable in a vegetarian diet than in a mixed Western diet (10% vs. 18%, respectively) (45, 46). Similarly, absorption of fat-soluble vitamins such as vitamin D and vitamin E, carotenoids, and fatty acids can be inhibited in diets high in fiber, including pectins and lignins (11). Vitamin B_6_ commonly is found as pyridoxine-β-D-glucoside in plants. This form has a bioavailability of approximately 50%, in contrast to free pyridoxine HCl, which is nearly 100% bioavailable in fortified foods (22, 47).

The lower bioavailability of vitamins from plant vs. animal sources has important ramifications. A number of scientific and policy organizations now advocate for an overall reduction in the consumption of animal-based foods, both for health and environmental sustainability reasons (10, 48, 49). However, micronutrient bioavailability in plant-based foods needs to be considered. Supplementation and food fortification with vitamins represent low cost, safe, and effective strategies to ensure adequate intakes of highly bioavailable vitamins in all diets, including in vegetarian or vegan diets (18, 49–54).

#### 3.1.3 Form of the nutrient

Several vitamins come in multiple forms, or vitamers, and the form that is ingested can have an impact on bioavailability. For example, the most common forms of vitamin A in the human diet are preformed vitamin A (retinyl esters) and pro-vitamin A (β-carotene) (46). The absolute bioavailability of β-carotene has been reported to be around 8–10% for cooked carrots, 15% for sweet potatoes, and 5% in spinach (10). The absorption and conversion of all-trans-β-carotene in oil is estimated to lead to a bioavailability of 40%, and for pure retinol of 80% (10, 46).

The EFSA NDA Panel concluded in 2014 that the bioavailability of food folate is around 50%, i.e., half that of folic acid taken on an empty stomach, whereas the bioavailability of folic acid from fortified foods or from a supplement ingested with food is about 85% (55). Indeed, Dietary Folate Equivalents (DFEs) have been established by national and international expert bodies to reflect these findings—1 μg of DFE is equivalent to 1 μg of food folate, but only 0.6 μg of folic acid either from a fortified food or a dietary supplement consumed with food (25, 28). The earlier IOM (NAM) equivalents are similar, but make a minor distinction in relation to folic acid on an empty stomach: 1 μg of DFEs is equivalent to 1 μg of food folate, or 0.5 μg of folic acid taken on an empty stomach, or 0.6 μg of folic acid with meals (25). Similarly, in Europe EFSA has established a conversion factor for calcifediol as 2.5 times the bioavailability of cholecalciferol, thereby recognizing the variable bioavailability across various vitamin D forms (56).

#### 3.1.4 Dose and stability

**Figure 2:**
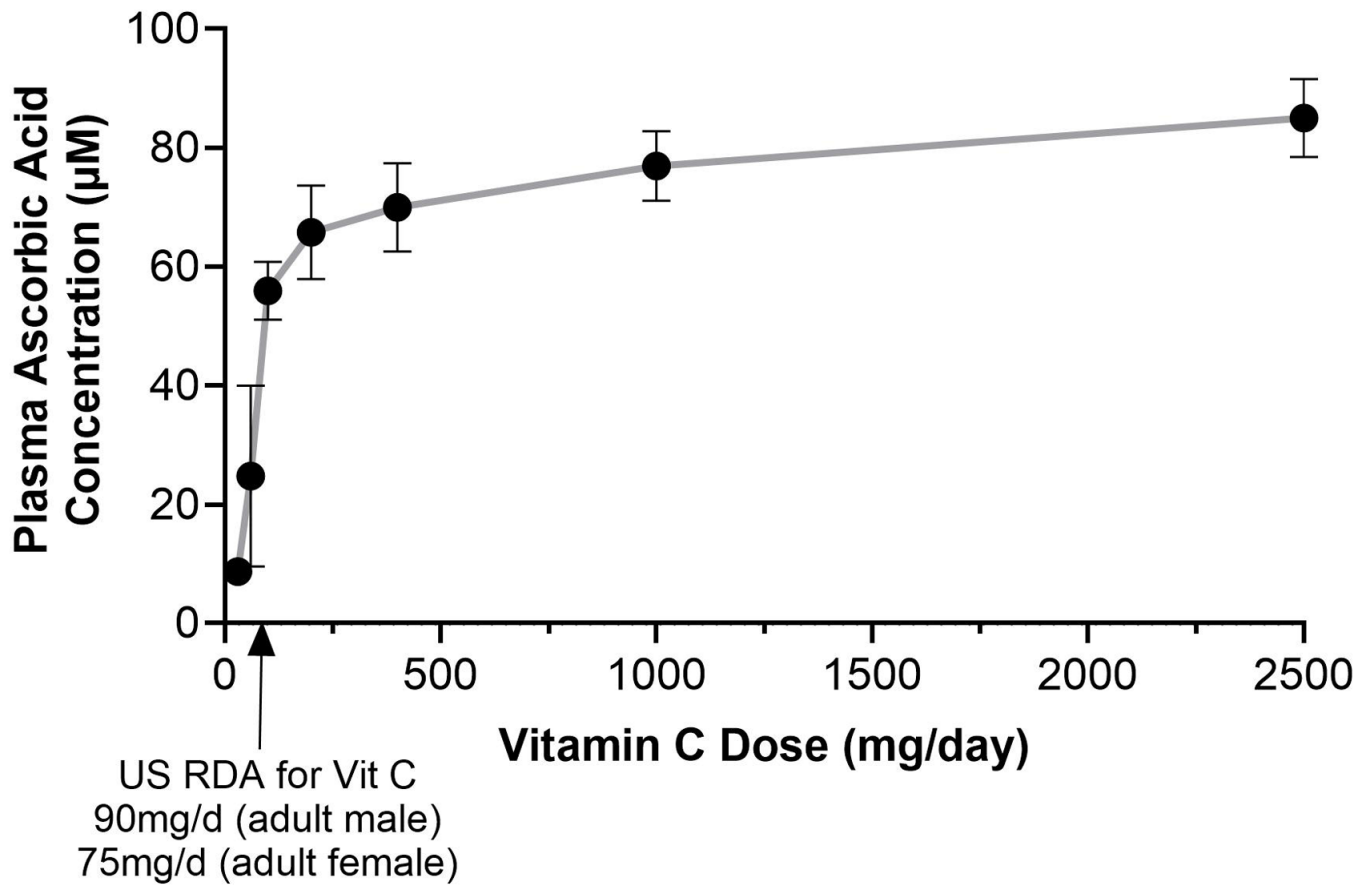
Bioavailability of vitamin C as a function of dose. The dose response of plasma ascorbic acid as a function of intake. The dose response is steep up to approximately 100 mg per day of intake, and then reaches a plateau although plasma concentrations still increase with increasing doses. Plasma ascorbic acid levels were well below the peak at intake levels corresponding to the US RDA for males (90 mg/d) and females (75 mg/d). Figure was created using data from (58).

Bioavailability of a nutrient can also depend on the intake of the nutrient. Vitamin C illustrates this well because its absorption is saturable. Clinical data indicate that between 80 and 100% of vitamin C at single doses up to 100–200 mg are fully absorbed into the bloodstream (57–59). However, bioavailability diminishes at higher doses, and is less than 50% at doses exceeding around 1,000 mg (58). At doses between 30 and 100 mg per day there is a steep, dose-dependent increase in circulating vitamin C levels, with saturation in the plasma at doses of 200–400 mg per day (Figure 2). Certain technologies, such as lipid-based formulations have been reported to increase the absorption into the bloodstream, both with respect to peak levels and total absorption as indicated by area under the curve (see below) (60).

Zinc also provides an illustrative example. As the content of absorbable zinc in the diet increases, the proportion of this zinc that is taken up decreases. This modulation of absorption efficiency is an important mechanism by which the body maintains zinc homeostasis (21, 61). Similarly, homeostasis also is maintained by variable rates of zinc excretion, and thus the balance of absorption and excretion. Indeed, substantial increases in absorbed zinc can be counterbalanced by increases in intestinal zinc excretion (61).

While not strictly a bioavailability issue, micronutrient stability can affect the outcome of micronutrient interventions if the consumer does not ingest the intended or labeled quantity of micronutrients. Vitamins are variously sensitive to heat (for example during cooking, pasteurization, or sterilization), moisture, oxygen, oxidizing and reducing agents, metallic ions, pH and other food components in different degrees (62). In addition to heating, other aspects of food processing can be destructive. For example, slicing or dicing of fruits and vegetables can increase the losses of vitamin C due to increasing the surface area of the food that is exposed to oxygen. Dehydration can lead to increased losses of vitamin C, niacin, and thiamin in vegetables. Other considerations of the food matrix itself can also be important. For example, thiaminase enzymes are present in certain foods including seafood and beans and can promote degradation of thiamin. On the other hand, the presence of egg albumin and casein enhance thiamin stability. All of these factors conspire to impact the stability of vitamins during processing and over the shelf-life, often necessitating the addition of an “overage” of vitamins during the formulation of fortified foods (62). These are important considerations—a wheat flour and maize meal fortification program of excellent design brought little impact due to insufficient dosing of the premix and perhaps stability issues as well (63). As vitamins can be formulated to withstand specific conditions in particular foods, there is an urgent need for the establishment of quality as a critical driver for effectiveness of food fortification programs.

### 3.2 Host-related factors

In addition to dietary factors, host factors also impact the bioavailability of nutrients. These can include age, gender, physiological state (for example, pregnancy, menstruation, lactation, or organ function), nutrient status of the individual, or the existence of certain disease states.

#### 3.2.1 Life stage

With respect to life stage, the maturation of the digestive tract and the concomitant absorption of nutrients (perhaps especially macronutrients) is still developing in infants in the first weeks and months after birth (11, 64). The elderly also experience differences in nutrient absorption when compared to younger adults, because of changes in secretory and absorptive capacity in the stomach or intestines that occur with aging. These changes do not impact macronutrient bioavailability, but do reduce the bioavailability of specific micronutrients, including certain B vitamins, vitamin D, and (primarily as a result of problems with vitamin D metabolism) calcium (11, 65, 66). Vitamin B_12_ malabsorption and deficiency are also common in older adults (67). Indeed, it has been estimated that between 20 and 40% of older individuals are deficient, with up to 70% of those cases due to malabsorption issues (68). Vitamin B_12_ absorption is mediated by its binding to intrinsic factor (IF), which is a protein secreted by parietal cells in the stomach. B_12_-IF complexes are bound by receptors in the ileum, in the presence of calcium, for absorption (67). The elderly are vulnerable to reduced IF secretion due to loss of parietal cells from pernicious anemia or atrophic gastritis. Decreased production of stomach acid and proteases are also common, and can lead to decreased release of B_12_ from ingested food proteins and thus decreased absorption (69). Similarly, common medications such as proton-pump and H2-receptor blockers also reduce gastric acid production, again reducing B_12_ bioavailability (68). For this reason, certain experts recommend supplemental vitamin B_12_ in older adults significantly exceeding published requirements (67).

#### 3.2.2 Physiological state, metabolic pathway status and hormonal influence

Physiological adaptations can occur in response to states of rapid growth, pregnancy, and lactation to increase the bioavailability of nutrients in response to elevated requirements. In addition, these changes can occur in the face of prolonged low nutrient intakes (11). These changes include increased absorptive capacity in the intestines and increased renal nutrient retention, among other mechanisms (11). These adaptations can be reversed when nutrient requirements decrease, or nutritional intakes increase.

Pregnancy and lactation provide examples of physiological states with increased nutritional requirements. Data indicate that calcium, zinc, iron, and copper intestinal absorption increases, and/or renal conservation increases during pregnancy and/or lactation (11). These effects appear to be at least partly due to the hormonal changes associated with pregnancy, particularly estrogen. Indeed, estrogen has been reported to regulate intestinal calcium absorption in humans and rodent models, with estrogen stimulating increased expression of calcium transport proteins in the duodenum (70, 71). Likewise, the increase in circulating copper concentrations observed during pregnancy has also been ascribed at least partly to increased estrogen levels (72).

Conversely, calcium absorption starts to decline during the transition to menopause, and circulating estradiol concentrations are reported to be a primary determinant of absorption rates (73). Furthermore, calcium absorption continues to decrease in postmenopausal women. These findings make sense, given the mechanistic studies described above. The health impact is enormous, as about a third of postmenopausal women will develop osteoporosis (74). Consistent with these data estrogen therapy improves calcium absorption, which is accompanied by increases in blood calcitriol, in postmenopausal women with osteoporosis (75, 76). For these reasons groups such as the NAM and the International Osteoporosis Foundation recommend increased calcium intakes in women over 50 years old compared to the same aged men or younger female adults (77, 78).

Apart from overall physiological status, the activation or inhibition of individual metabolic pathways across tissues and nutrient-induced transporter desensitization can also influence bioavailability (79). A prominent example is the secretion of hepcidin following iron exposure in the GI tract, which acts to inhibit further iron uptake via its influence on the cellular iron exporter ferroportin (discussed in more detail below) (80).

#### 3.2.3 Disease states and medications

A wide variety of disease states can alter nutrient bioavailability and/or status. Diabetes is one notable example. Metformin is the first-line drug prescribed for glycemic control in type 2 diabetes mellitus (T2DM) and is reported to be among the top 10 of medicines prescribed globally. Although metformin has a good safety profile, its use is well-described to be associated with an increase in vitamin B_12_ deficiency, at least partially by reducing B_12_ absorption in the small intestine (81, 82). Metformin-induced B_12_ deficiency is likely a cause of diabetic neuropathy, which can affect up to half of type 2 diabetics (81). Metformin may also contribute to reduced status of other B vitamins, including thiamine and folate, as well as potentially vitamin D and magnesium. Furthermore, a substantial percentage of type 2 diabetics are prescribed medications including proton pump inhibitors and histamine H2 receptor antagonists, both of which also negatively impact vitamin B_12_ and magnesium status (81). Vitamin B_12_ supplementation has shown to be effective at preventing or treating metformin-induced B_12_ deficiency, and potentially at alleviating neuropathy (82, 83).

Cancer is among the leading causes of death globally, and has substantial impact on nutritional status (84, 85). Indeed, malnutrition is a serious cancer co-morbidity that impacts between 40 and 80% of cancer patients, negatively influencing quality of life, numerous treatment outcomes, and survival rates (86). The malnutrition can result from systemic inflammation that leads to reduced food intake (anorexia) and ultimately weight loss. Other contributing factors to reduced intake include alterations in taste and smell perception, mouth sores, early satiety, nausea and vomiting, swallowing difficulties, reduced intestinal motility, and side effects from medications (86, 87). Due to reduced intake, the bioavailability of the nutrients that are ingested is critical. Unfortunately, malabsorption is also common in these patients (87). Malnutrition in these patients contribute to sarcopenia and cancer cachexia, although cachexia is not treatable by conventional nutritional support alone. Indeed, the most pressing nutritional problem in cancer is muscle loss, which predicts negative outcomes including decreased survival. Micronutrient status and supply can also be a concern (86). For these reasons, regular nutritional screening for cancer patients is recommended, including assessment of nutritional intakes, weight changes, and BMI (87, 88). Both the 2017 and 2021 ESPEN guidelines for clinical nutrition in cancer patients recommend nutritional interventions, including oral nutritional supplements, for those who can eat but are at risk of malnutrition. These guidelines include a strong recommendation for protein intakes above 1 g/kg/d and up to 1.5 g/kg/d, and a strong recommendation that “vitamins and minerals be supplied in amounts approximately equal to the RDA” (87, 88). Use of micronutrient supplements is also supported by the American Cancer Society and the American Institute for Cancer Research (86).

Furthermore, a wide variety of common medications can impact nutrient bioavailability or status (89). As mentioned above, proton pump inhibitors reduce absorption of dietary (but not supplemental) vitamin B_12_ and may also reduce calcium uptake. Certain oral contraceptives appear to reduce the status of many vitamins and minerals, including folate (90). Certain classes of anti-epileptic drugs reduce folate absorption and increase folate metabolism (91). Diuretic use increases urinary excretion of magnesium and potassium (89). Methotrexate, which is a cancer drug and is also used to treat autoimmune diseases, is a folate antagonist (91). Likewise, statins are reported to reduce vitamin D synthesis, because vitamin D is synthesized by the body from cholesterol (92). Unsurprisingly, certain medications that reduce absorption of fat (often as a treatment to support weight loss) also reduce the absorption of fat soluble vitamins, including vitamins A, D, E, and K (93). Additionally, glucagon-like peptide-1 (GLP-1) modulating drugs which have become widely used, reduce micronutrient absorption through reduced food intake and gastrointestinal effects (see below). These micronutrient-drug interactions can result in micronutrient inadequacy or deficiency, especially with long term medication use, and in many cases can necessitate dietary supplementation.

#### 3.2.4 Nutritional status and nutrient-nutrient interactions

A classic example of the impact of nutritional status on nutrient bioavailability occurs with iron. The human body regulates the absorption of iron such that absorption diminishes as iron status improves (94). In addition, in the case of iron inadequacy, other mechanisms allow the “export” from iron stores to regulate the status (95). Plasma iron concentrations and total iron content of the body are regulated by hepcidin, which inhibits the iron transporter ferroportin to regulate iron absorption from the duodenum, and the release of iron from hepatocytes and macrophages. Inhibition occurs by direct binding, as well as inducing ferroportin ubiquitination, endocytosis, and degradation. Hepcidin production by hepatocytes is regulated by status—an iron deficient individual will produce less hepcidin, and one who is replete will produce more. Notably, serum hepcidin levels are reported to decrease during menstruation, and subsequently increase during the second half of the menstrual cycle (96).

Hepcidin production also is regulated in pregnancy in order to meet the increased demand for iron from both the mother and fetus (80). It has been elegantly demonstrated that iron supplementation increases hepcidin levels, whereby the iron supplementation paradoxically drives reduced iron bioavailability, and that therefore alternate day supplementation is more effective than daily supplementation, showing that also timing of intake can influence bioavailability of nutrients (97).

The status of one (or several) micronutrients can also affect the bioavailability of others. For example, in humans, vitamin A deficiency can impair intestinal iron absorption and mobilization, and vitamin A supplementation can enhance the efficacy of iron supplementation through the enhancement of absorption, mobilization and erythropoiesis (98). The net result is an improvement in hematological indicators. Vitamin C also enhances the absorption of dietary iron, influences its mobilization, and dampens the prooxidant effect of iron, together with vitamin E (98–100). Riboflavin enhances the hematological response to iron, and as such riboflavin deficiency may be a primary cause of anemia in many populations due to the condition of immature intestinal villi associated with riboflavin deficiency, and its role in iron mobilization (98). Adequate status of all these vitamins, along with folate, vitamin B_6_, vitamin B_12_, and potentially vitamin D, plays roles in reducing the incidence of anemia (101). The anemia example, demonstrating the interdependency of multiple micronutrients in iron metabolism, highlights the importance of a holistic approach to nutrition, and that simply providing higher doses of a single nutrient (in this case, iron) is not the right strategy (98, 101).

#### 3.2.5 Microbiota

Understanding the contribution of the gut microbiota to nutrient bioavailability is still an emerging area of research, but it is clear that commensal species exert an impact on vitamin supply and bioavailability to the host. The so-called “micronutrient-microbiome axis” operates in both directions—the microbiota both consume nutrients including vitamins in the gut, and produce them as well (102). As such, increasing evidence demonstrates that commensal species can both positively and negatively impact the amount and/or absorption and bioavailability of a wide range of vitamins and minerals (103, 104). For example, supplementation with vitamins A, B_12_, C, and D are reported to promote growth of beneficial species from a variety of genera, including Bifidobacterium, Lactobacillus, and Roseburia. Certain bacterial genera, including Bifidobacterium, Enterococcus, and Bacteroides synthesize substantial amounts of both B and K vitamins (102, 103, 105). Moreover, B vitamin absorption may be enhanced by probiotics that regulate intestinal pH (104). Associational studies suggest a relationship between the gut microbiota and fat soluble vitamin status, even though the microbiota are not known generally to produce most of these vitamins (vitamin K being an exception) (103). Finally, there are also reports that supplementation with certain probiotic strains can lead to an increased vitamin status. For example, supplementation with a *Lactobacillus reuteri* strain was associated with an increase in vitamin D status (106).

Increased bioavailability of minerals seems to be related to the availability of bacterial enzymes or production of short-chain fatty acids (SCFA) by the microbiota. Bacterial phytase may be capable of breaking down chelated complexes with iron, calcium, magnesium and phosphorous, thus making these minerals available in the lumen. Human studies have shown that iron absorption is enhanced when providing specific probiotics (102). In animal studies, a higher production of SCFA appears to facilitate calcium and phosphorus absorption. In human studies, supplementation with a prebiotic increased calcium absorption; the postulated mechanism is a decrease in pH due to higher microbiota activity which facilitates minerals remaining in their soluble form (102). Finally, zinc bioavailability increases when combining supplementation with certain probiotics (103).

Dysbiosis can negatively impact nutrient bioavailability. Small intestinal bacterial overgrowth (SIBO) is a condition that exemplifies this. SIBO is characterized by both an increase in the amount of bacteria in the proximal small intestine, as well as a change in the bacterial profile of certain groups such as coliforms, more commonly seen in the distal intestine (107, 108), with varying degrees of severity (107). SIBO is reported to be accompanied by villous atrophy and inflammation of the intestinal epithelium, resulting in decreased absorptive surface area and capacity, deconjugation of bile acids by the microbiota leading to malabsorption of fat and fat-soluble vitamins, bacterial degradation of sugars, and reduced activity of brush border enzymes (108). From a micronutrient perspective, SIBO is linked to reductions in bioavailability (and increased risk of deficiency) in vitamins A, D, E, B_12_, as well as iron (107, 108).

## 4 Relevance of bioavailability in the current nutritional context

### 4.1 Poor quality diets

The bioavailability of nutrients in the diet, or from supplements, is important given the poor nutritional status common around the world today. Survey data consistently indicate widespread nutrient inadequacy and deficiency in a wide variety of vitamins and minerals, both in vulnerable subpopulations and the general population as a whole (5, 12, 109). Diets of low-income populations not only are monotonous and tend to lack essential nutrients but are also often rich in inhibitors. For example, in 2021 in Africa, 74% of caloric intake came from wheat, rice, maize, starchy roots, sugar and sweeteners, and vegetable oil, all of which are very poor in micronutrients in their refined and unfortified forms (110). In addition, these populations also often have impaired nutrient absorption capacity, due to physiological changes deriving from poor nutrient status and high prevalence of infectious diseases (18).

Consumption of diets that are not well-balanced is also prevalent in high-income countries. As one example, a survey of dietary habits of 2,057 adults in Switzerland compared intakes and assessed them for compliance to the food-based dietary guidelines (FBDG) of the Swiss Food Pyramid (111). Less than 1% of the population followed all seven FBDGs, and only 41% met at least three of the seven. While regional (German, Swiss, or Italian-speaking areas) differences existed, in general the Swiss overconsumed in the categories of sweets, salty snacks & alcohol; added animal fats; and meat. They under consumed fruit & vegetables (only 18% met the recommended intake), dairy, and cereal products and potatoes. Not surprisingly given these intake data, the Swiss adult population, and especially the elderly population, is reported to be at risk for deficiencies in many nutrients including vitamins C and D, iron, selenium, and zinc (112). The situation may be even less favorable if bioavailability-driven nutrient status was assessed. These data clearly illustrate that access to a healthy diet is not sufficient to guarantee healthy nutrient status, undermining the popular paradigm that supplementation has no value if a healthy diet is available.

### 4.2 Vegetarian and vegan diets

As noted above, vegetarian and vegan diets are increasingly recommended and embraced for numerous reasons including their health benefits and for environmental sustainability reasons (49). However, one must take care to consume adequate amounts of micronutrients as certain vitamins, and minerals such as iron and zinc from plant-based diets can be less bioavailable than from diets that include animal-based foods (10, 49). The micronutrient content of cereal grains and legumes are also diminishing because of climate change (see below).

Several dietary modeling studies have been published investigating the potential impact of vegetarian or vegan diets. While the results of each model vary, switching from a diet incorporating animal-based foods to a diet with an increased percentage of plant-based foods, or exclusively plant-based foods has been predicted variously to increase the risk of inadequate intakes of several vitamins (A, B vitamins especially B_12_, D), minerals (calcium, zinc, heme iron), and choline (113–116).

Recent cohort studies confirm that shortfalls in intake and/or status of many of these nutrients is common in vegetarians and/or vegans, and additionally iodine in vegans (49, 117–122). The WHO has stated that vegan diets are associated with low intakes of vitamins B_2_, B_12_, and D, as well as zinc, calcium, and selenium, and that vegans “may consider the consumption of fortified foods” (49). Vitamin B_12_ is of particular focus since this vitamin is found almost exclusively in animal products (120). It should be noted, however, that these shortfalls are not unique to vegetarians and vegans, and in fact are also common in those that consume non-vegetarian diets as well. Indeed, obtaining recommended intakes and status of micronutrients is certainly attainable with carefully-designed plant-based diets (49, 123). Thus, vegetarians and vegans (as with the rest of the population) are advised to select diets that are high in bioavailable micronutrients and/or low in inhibiting factors and complement their diets with supplements and fortified foods as needed, with particular focus on vitamin B_12_ (49, 123).

### 4.3 Decreasing rates of micronutrient intake

In addition to the challenges described above with suboptimal diet quality and reduced micronutrient bioavailability associated with plant-based diets, it is concerning that the actual intake of micronutrients is decreasing in some populations, including in high income countries. As one example, a recent study reports that the intakes of vitamin C in Korea decreased by 51% between 1998 and 2018, to 61 mg/d, which is below published requirements for vitamin C intake in adolescents and adults (6, 124, 125). This decrease is linked to an overall reduction in the intake of fruits, vegetables, and grains in Korea over this same time. Similarly, in the United States, vitamin C consumption also decreased over essentially the same timespan (1999–2018), by 23% to an average of 75 mg/d (7). Like the situation in Korea, this decrease was driven by a near 50% reduction in 100% fruit juice, the leading source of vitamin C in the U.S. While this average intake does meet the US RDA, the percentage of the population that failed to achieve the EAR increased from 38% in 1998 to 47% in 2018, with certain subpopulations reaching more than 60% below the EAR. The most prominent increases in the percentage below the EAR included toddlers, those 14–18 years of age, and males that were 19–50 years or ≥71 years (7). Since 2002, U.S. infants and toddlers have also seen concerning decreases in intakes of vitamins C and D, as well as iron in 6–11.9 month old infants due to decreased consumption of iron-fortified infant cereals (126, 127). The authors of these studies suggested that increased consumption of meats and fortified cereals designed for older infants could help increase iron intakes, and that dietary supplements likely could also improve the situation. In China between 1989 and 2015, intakes of several micronutrients have decreased among adults aged 18–35, including in particular vitamins A, B_1_, and C (8). Finally, a recent report concludes that 1 in 5 Europeans are now devoting less of their budgets to purchase of supplemental vitamins and minerals (128).

The concentration of essential nutrients in fruits and vegetables is also declining over time. A reduction of 5–40% in vitamins and minerals has been reported when examining their content in the past decades. This can be due to a “dilution effect” where larger crop yields imply lower nutrient concentrations, a narrow selection of plant varieties for yield and resistance reasons, and mineral depletion of soils (129–131). Indeed, micronutrient deficiencies are routinely seen in intensively grown vegetable crops, pulses, cereals, and oilseeds including in India (126). This implies that soil or foliar application of minerals could improve the situation (131). A recent study reports that foliar application of zinc can dramatically increase the zinc content of finger millet, to a greater extent than soil application, although soil application was more effective at increasing yield (132). However, some have expressed concern about the usage of mineral fertilizers, due to their expense and sustainability concerns among others, and instead promote organic amendments or other strategies such as biofortification (131, 133). A recent publication explored the use of dewatered sewage sludge (SS) and poultry manure (PM) as alternatives to mineral fertilizers. This group reported that PM significantly increased plant zinc although it decreased with SS and there was no impact on plant copper. Both amendments improved plant growth and soil fertility (133).

Furthermore, climate change and its drivers also are diminishing the nutritional composition of crops, and this impact is predicted to intensify as atmospheric CO_2_ levels increase. Increasing atmospheric CO_2_ leads to decreases in the concentration of protein and minerals (e.g., iron and zinc) in cereal grains and legumes, and B vitamins in rice (134–136). Declines in nutrient content are predicted to place hundreds of millions of additional people to be at risk for zinc, iron, and/or protein deficiency, in addition to the 2 billion people who are deficient in zinc or iron today (134, 136).

Overall, most of the global population fails to consume adequate amounts of micronutrients from the diet (excluding fortification and supplementation). As mentioned above, a recent study estimates that at least 5 billion people worldwide have inadequate dietary intakes of iodine, vitamin E, or calcium; 4.9 billion do not consume enough iron; and more than 4 billion do not take in enough riboflavin, folate, or vitamin C (4). Likewise, billions have inadequate intakes and/or deficiencies for one or more of vitamins A, B_6_, B_12_, D, thiamin, niacin, selenium, magnesium, and zinc (4, 5, 137–139).

### 4.4 Obesity

Worldwide obesity has nearly tripled since 1975 (39% of adults aged 18 years and over were overweight in 2016, and 13% were obese). Indeed, most people live in countries where mortality from overweight and obesity exceeds mortality from being underweight (140). Despite excessive dietary consumption (energy-dense but nutrient-poor foods), obese populations have high rates of micronutrient deficiencies. Indeed, observational data show an inverse association between BMI and nutritional status for vitamin D, iron, vitamins B_1_, B_6_, B_12_, folate, vitamin A, vitamin C, magnesium, selenium, zinc and potassium (141–148).

The demand for micronutrients could be altered due to their condition. For instance, the increased body mass in individuals with obesity may necessitate a higher intake of nutrients to compensate for partitioning between lean and fat mass and metabolic and microbiota alterations (149–151). Obesity is linked to persistent low-grade inflammation and heightened oxidative stress, resulting in an increased demand for antioxidants and specific micronutrients (149). The inadequacies in certain micronutrients seen in obesity, such as vitamins C and D and zinc, could potentially contribute to insulin resistance (149, 151). Additionally, the expanded volume of body fluids and body fat mass in obese individuals may induce a dilution effect, causing lower concentrations of certain nutrients in the bloodstream and requiring elevated intake to sustain optimal levels (149, 151–154).

GLP-1 is one of several gastrointestinal hormones that influence the control of eating and satiety, gastric emptying, meal-related glycemia, and weight gain. GLP-1R agonist drugs promote satiety, thereby reducing food intake and leading to weight loss (155, 156). Historically, these drugs were prescribed to improve glycemic control in type 2 diabetic patients, but more recently they have become popular as weight loss drugs particularly for obesity. Indeed, U.S. doctors wrote more than 9 million prescriptions for GLP-1R agonist medications during the last 3 months of 2022 (157). Data on micronutrient status of patients taking GLP-1R agonist drugs are lacking. Given that the obese population already suffers from micronutrient deficiencies, and that these drugs reduce food intake and can induce gastrointestinal side effects such as diarrhea and vomiting, it is reasonable to expect that micronutrient intakes in this population would remain poor. Thus, supplementation or food fortification with a range of highly bioavailable micronutrients may be critical for this population.

## 5 Micronutrient intervention strategies

Given the above, it is generally accepted that vitamin and mineral inadequacies and deficiencies are widespread globally. While a thorough review of public health intervention strategies to address these challenges is beyond the scope of this review, a few are worth mentioning. The major tools available in the fight against micronutrient malnutrition are dietary supplementation, food fortification, and dietary diversification (a food-based approach). Each has its strengths and limitations and domain of applicability for improving micronutrient status in a population over varying periods of time (18, 158).

Strengths of dietary supplementation include its rapid impact on micronutrient status, health, and life expectancy of the individuals consuming the supplement. Historically supplementation approaches have been utilized for vitamin A and iron deficiency. One example is a partnership between Nutrition International, UNICEF and other groups that has provided over 10 billion vitamin A capsules to children under 5 years old (two annual doses per child), in 55 countries to prevent blindness and improve immunity (159). A key limitation of supplementation is that periodic high coverage has often not been sustained over time for a variety of reasons, including lack of sustained financial or political support or other overriding priorities and poor compliance by the target individual (158).

Experience has shown food fortification to be effective and a useful bridge to sustainable, long-term dietary change. If the program is universal through a commonly consumed product, fortification implies little creation of consumer demand, fewer logistical problems in supply than supplementation and its low costs are borne almost exclusively by the consumer. It has the potential to have a broader impact when compared to supplementation, and its impact is evident in the short to medium term (158). In addition, when consumed regularly fortified foods can maintain body stores of nutrients more effectively than intermittent supplementation, and it can be highly cost effective (18, 160). Examples of successful fortification campaigns include the fortification of liquid dairy products and fat spreads in Finland with vitamin D and the fortification of folic acid into flours to reduce incidence of neural tube defects (51, 52). Success of this strategy of course depends on adequate fortification rates of micronutrients to the staple food, as demonstrated in a fortification campaign in South Africa (63).

Food-based approaches attempt to correct the underlying causes of micronutrient deficiencies by encouraging changes in dietary habits of the population within their cultural context. These strategies are usually considered the ideal long-term goal. Nevertheless, experience has shown that while dietary modification can be brought about by nutrition education, to even begin to achieve these changes may require 5–10 years or more, assuming a stable economic and political environment, and modifications in agricultural activities —such as crops raised and food production— as well as on food marketing, preservation, and preparation (158).

## 6 Case studies: vitamins and forms

The literature indicates that vitamins added to foods or taken as supplements generally are at least as bioavailable as endogenous ones, and often more so since those in foods may be bound to the food matrix (10, 21, 26). For example, as described above the bioavailability of vitamin A, niacin, riboflavin, vitamin B_6_ and vitamin B_12_ is reduced by the presence of fiber (161). The bioavailability of supplemental folic acid is substantially greater than that of naturally occurring folate in food (10, 25). Indeed, food folate is less bioavailable than folic acid with a median relative bioavailability of 65% (range: 44–80%), an estimate that approximates the 60% value derived from the DFE equation (162). Similar findings have been reported for supplemental vs. endogenous vitamin B_6_, B_5_, β-carotene, and vitamin K, particularly when comparing supplemental vitamins to those in plant-based diets. The bioavailability of added vitamin A esters is generally high and is typically not strongly influenced by the food matrix (163). All steady state comparative bioavailability studies in humans have shown no differences between synthetic and natural vitamin C, regardless of the subject population, study design or intervention used (26).

As such, the bioavailability of added vitamins is generally high. Nevertheless, a number of technologies are available to increase micronutrient bioavailability that can be implemented on an individual or population basis.

### 6.1 Vitamin D and calcifediol

As described above, many vitamins occur in the diet in different forms (vitamers), that can impact bioavailability or bioactivity. Vitamin D is a good example of this. Dietary vitamin D occurs as vitamin D_3_ (primarily from fish, meat, and eggs), vitamin D_2_ (primarily from mushrooms and yeast), and to a lesser extent, calcifediol. All three forms are available as dietary supplements. Calcifediol is the 25-hydroxylated form of vitamin D_3_ (25(OH)D) and is the primary form of vitamin D in the bloodstream. It is present in the diet, predominantly in meat, dairy, eggs, and in some markets as a dietary supplement (56, 164, 165).

**Figure 3:**
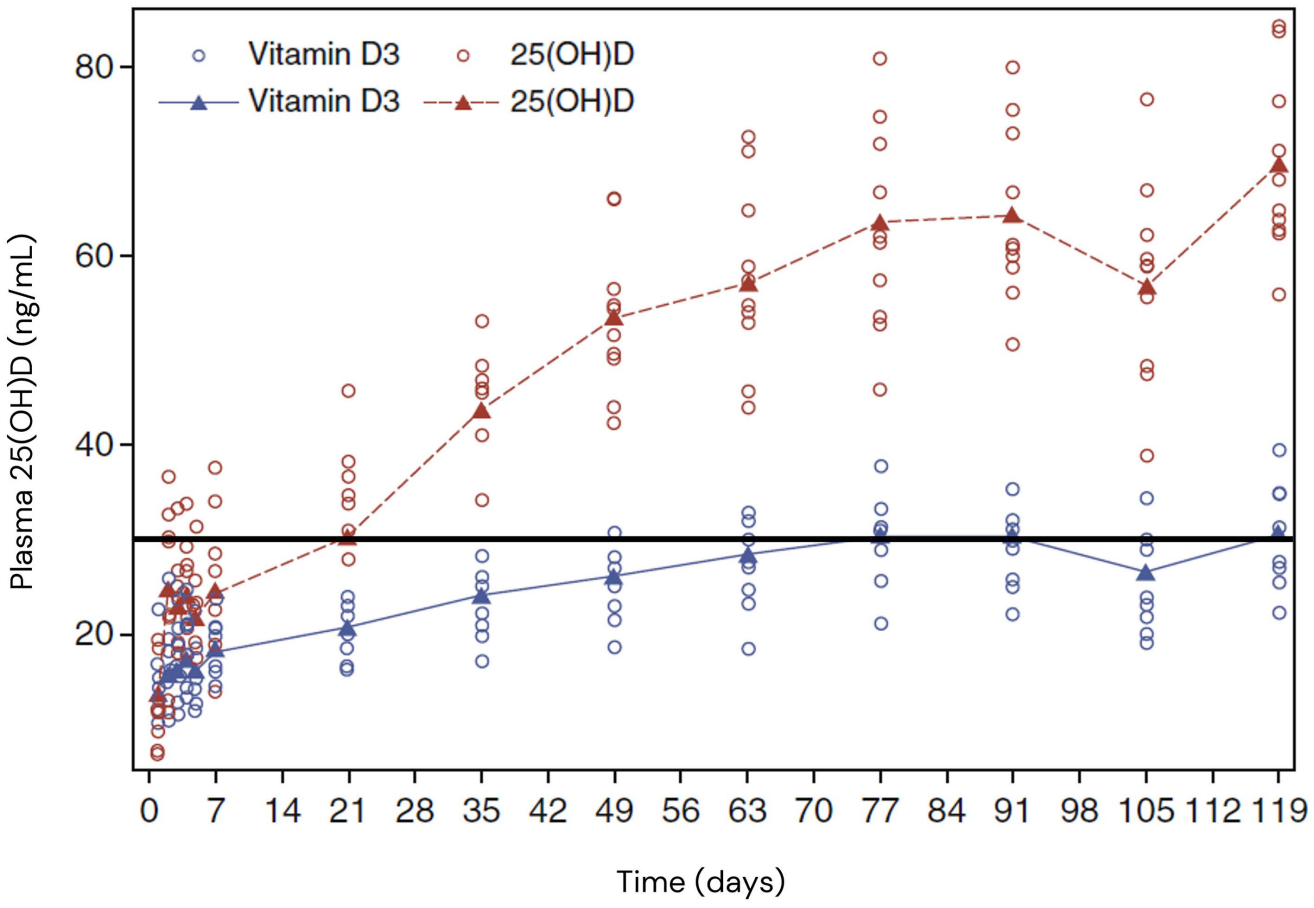
Calcifediol is faster and more effective than vitamin D3 at raising serum 25(OH)D status. The graph shows individual participant (colored circles) and group model-predicted mean data (colored lines) (172). Healthy postmenopausal women took 20 μg/d of calcifediol (25(OH)D, red circles) or vitamin D3 (blue circles). Serum 25(OH)D levels were measured on 14 clinical visits through 4 months of intervention. Vitamin D sufficiency (30 ng/mL (75 nmol/L) 25(OH)D) is indicated by the solid black line. Each group mean reached the 30 ng/mL threshold (red dashed line and triangles for calcifediol, blue line and triangles for vitamin D3), although the calcifediol group reached this threshold more rapidly. While all participants taking calcifediol reached sufficiency by 35 days, the authors reported that about 50% of those taking vitamin D3 did not reach sufficiency over the entire course of the study. Used with permission of Springer Nature BV, from Chapter Relative Effects of Vitamin D3 and Calcifediol, Bischoff-Ferrari et al., 2013 (172); permission conveyed through Copyright Clearance Center, Inc.

Vitamin D insufficiency and deficiency are common globally. Supplementation with vitamin D_3_ or D_2_ is a safe and inexpensive strategy to improve vitamin D status. However, optimizing serum 25(OH)D levels with these vitamers can take relatively long periods of time (weeks or months), with a substantial percentage of individuals supplementing at recommended levels not reaching an adequate status, especially elderly or overweight people (Figure 3) (166–172). Scientific assessment of clinical trial data and recent expert review indicate that at physiological intakes, calcifediol is approximately 3× more effective than vitamin D_3_ at raising blood vitamin D levels above baseline status. Furthermore, the clinical data indicate that at equivalent doses, those taking calcifediol reached adequate status (75 nmol/L or 30 ng/mL 25(OH)D) more quickly and reliably than those taking vitamin D_3_ (Figure 3 and Table 2) (154). Indeed, a greater percentage of study participants taking calcifediol achieved sufficiency than participants taking vitamin D_3_ (Figure 3). In the EU, EFSA has concluded a 2.5× conversion factor “reflects the relative bioavailability of calcifediol” as compared to vitamin D_3_, when used in food supplements (56). There is compelling evidence that the ability of calcifediol relative to vitamin D_3_ to increase vitamin D status is even greater in those with obesity, possibly due to a variety of factors including increased sequestering of vitamin D_3_ in fat stores (150, 173).

**Table 2:** Relative effectiveness and speed of calcifediol vs. vitamin D_3_ at raising circulating 25(OH)D levels in clinical subjects.

Reference	Source	Dose (μg/d)	Time to reach 75 nmol/L (days)^a^	Relative speed to reach 75 nmol/L (days)^b^	Relative effectiveness in raising serum 25(OH)D levels^c^
(206)	D_3_	25			1
	calcifediol	10			3.5
		20			3.3
		50			3.5
(166)	D_3_	20	70 (69 nmol/L)	1	1
	calcifediol	7	70 (71 nmol/L)		4.2
		20	35	2.0	5.0
(168)	D_3_	20	68	1	1
	calcifediol	20	17	4.1	2.8
(207)	D_3_	60	112 (74 nmol/L)		1
	calcifediol	20	28		5.5
(169)	D_3_	20	112		1
	calcifediol	5	not reached		1.0
		10	56		3.0
		15	28		2.8
(170)	D_3_	20	40	1	1
	calcifediol	10	26		3.0
		15	10		2.9
		20	7	5.7	2.8
Average				3.9	3.3

a The time (days) for the mean serum 25(OH)D level in the study population to reach sufficiency (75 nmol/L) is indicated, when it was reported in the individual study. In some instances, the mean did not quite reach 75 nmol/L (as indicated). In the case of (166), those taking vitamin D_3_ achieved a serum level of 69 ± 8.7 nmol/L at 70 days. In this case, 70 days was taken as the time to reach sufficiency for the purposes of estimating a relative speed factor.

b Relative speed is defined as the time needed for the study participants taking calcifediol to achieve a mean serum 25(OH)D level of 75 nmol/L, as compared to those taking an equal dose of vitamin D_3_. Values were calculated as days to reach sufficiency in the vitamin D_3_ group divided by the days to reach sufficiency in the calcifediol group. Comparisons of unequal daily doses were not considered.

c Relative effectiveness was calculated as the ratio of the difference in serum 25(OH)D (final minus baseline) in the group taking daily calcifediol vs. the group taking vitamin D_3_, corrected by the daily dose. Values are from (154) and (170).

From a mechanistic perspective, there is a marginal difference in absorption in favor of calcifediol vs. vitamin D_3_ in healthy individuals, although that difference is greatly enhanced in those with intestinal fat malabsorption issues (154). More importantly, unlike calcifediol, vitamin D_3_ requires a hydroxylation step in the liver to be converted to 25(OH)D and thus to raise vitamin D status. The existing data indicate that only a minority of vitamin D_3_ molecules are converted to 25(OH)D, especially at higher supplemental doses (154). Thus, when compared to vitamin D_3_, an increased proportion of ingested calcifediol becomes available for use in normal metabolic and physiological functions and processes, which is one of our definitions of bioavailability.

Calcifediol is approved for commercial use as a dietary supplement in several countries including Australia, New Zealand, Brazil, Singapore, and Malaysia. Recently, the European Commission has authorized calcifediol as a novel food and as a form of vitamin D for food supplements (174, 175).

### 6.2 Folate and methylfolate

**Figure 4:**
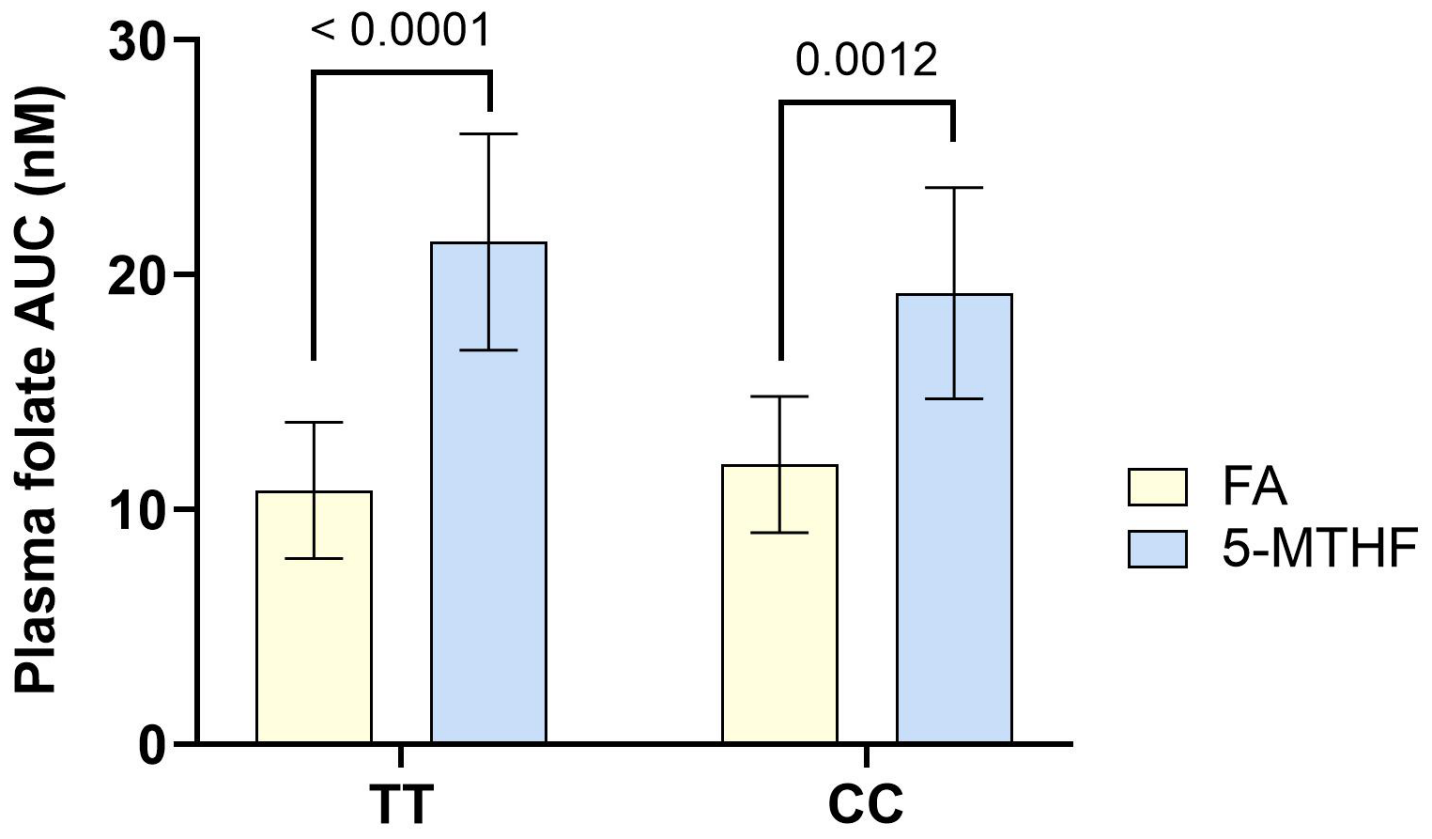
Bioavailability of MTHF is greater than that of folic acid in women with both wild type (CC) or mutated (TT) alleles of the MTHFR gene. Healthy women who were homozygous for either the wild type allele (CC) or 677C➔T mutation (TT) of the 5,10-methylenetetrahydrofolate reductase gene (MTHFR) were given a single dose of folic acid (FA, 400 μg) or methylfolate (MTHF, 416 μg) in a randomized crossover design. Plasma folate was measured at multiple timepoints through 8 h. The graph shows the mean AUC for total plasma folate concentration. p values for the comparisons between folate sources are shown above the bars. Figure was created using data from Prinz-Langenohl et al. (178).

There are primarily two forms of folate, folic acid and methylfolate (176). Folic acid is the synthetic form found in dietary supplements and fortified foods. After absorption, it must go through a series of enzymatic processes to convert to the active form, methylfolate. Depending on the population, up to approximately 60% of people have limited ability to convert folic acid to methylfolate due to mutations in the methylenetetrahydrofolate reductase (MTHFR) gene (177). Methylfolate is the form found naturally in foods, and since it is already in the active form it does not need to be converted. It is also more bioavailable and absorbed faster than folic acid, both in women with and without MTHFR gene mutations (Figure 4) (178). Absorption and bioavailability may be especially important for women of childbearing age, because achieving a blood threshold level of >906 nmol/L prior to the neural tube closure at 4–6 weeks of pregnancy is protective against neural tube defects (179). In a 12-week study, 142 healthy women of childbearing age who were supplemented with methylfolate had higher red blood cell folate levels compared to those supplemented with an equimolar dose of folic acid (1,951 ± 496 vs. 1,498 ± 580 nmol/L, *p* < 0.001) (180). However, folic acid is the form that was clinically shown to prevent neural tube defects and is the form recommended by the WHO and other expert groups (181, 182). Combining methylfolate with folic acid may be advantageous. In women of childbearing age, it took 8 weeks of supplementation with either 400 mcg of folic acid or the equimolar dose of methylfolate (416 mcg) to reach the protective blood folate threshold level (>906 nmol/L) (183). But when 400 mcg of folic acid was combined with 451 mcg of methylfolate, the protective blood threshold level was achieved in only 4 weeks (184). Methylfolate has also been reported to increase red cell folate levels to a greater extent than does folic acid in formula-fed infants (185).

Methylfolate is widely available as a dietary supplement and/or as a source of folate in infant formula in several regions including Europe, Asia, Latin America, and the United States.

## 7 Case studies: enhancers of bioavailability

Enhancing the bioavailability of nutrients is a critical aspect of nutritional science. As described above, certain diets or matrices can contain antagonists or other components that limit the absorption of nutrients. Also as mentioned, there can be endogenous limits on the amount of these nutrients that can be absorbed and/or retained by the body, independent of the existence of dietary antagonists. Nutritional innovation strategies to enhance delivery of nutrients can take advantage of several technologies that help solve for both of these situations, in order to optimize nutritional delivery and status, spanning from enzymatic treatments to specialized formulations and delivery methods (see Table 3). For instance, phytase, permeation enhancers, lipid-based formulations, and micronutrient compounding are at the forefront of bioavailability innovation. These technologies, specifically chosen for this review, represent solutions that target both exogenous barriers (such as dietary antagonists) and intrinsic absorption limits, ensuring efficient nutrient delivery across various dietary contexts. Although other bioavailability enhancers exist, these four represent the most impactful and practical for current nutritional formulations, optimizing nutrient absorption while maintaining safety and meeting regulatory standards in dietary supplements.

**Table 3:** Technologies to improve nutrient bioavailability.

Technology	Mechanism of Action	Benefits	Limitations	References
Phytase^*^	Breaks down phytic acid, reducing mineral binding and enhancing mineral bioavailability	Improves absorption of iron, zinc, calcium, and magnesium	Limited effect in non-plant-based diets	(208, 209)
Medium-Chain Fatty Acids (C8, C10)^*^	Paracellular (tight junction modulation), transcellular (membrane fluidization)	Effective for lipid-soluble vitamins and for peptides	Potential irritation in high doses	(190, 195, 210)
Eligen™ Technology (SNAC, 4-CNAB, 5-CNAC)^*^	Transcellular (carrier mechanism, pH elevation, gastric enzyme inhibition)	Improved peptide and vitamin bioavailability	Expensive, complex formulation requirements	(192, 196)
Bile Salts^*^	Transcellular (membrane fluidization)	Enhanced fat-soluble vitamin uptake	Can cause GI discomfort or diarrhea in some users	(211)
Lipid-Based Formulations (Liposomes)^*^	Protects nutrients from degradation, promotes sustained release and absorption via micelles	Effective for vitamins, protects against GI degradation	Requires complex production	(60, 212, 213)
Compounded Nutrients (Emulsified Beadlets)^*^	Reduces particle size for improved solubility and absorption	Highly effective, customizable	Specialized processing needed	(214, 215)
Piperine^*^	Increases transcellular uptake	Enhances nutrient absorption, widely used in supplements	Potential drug interactions	(216, 217)
Acyl-Carnitine	Transcellular (membrane perturbation), paracellular (tight junction modulation)	Effective for cellular uptake	Limited research in diverse nutrient types	(218)
Sucrose Laurate	Combined transcellular/paracellular action, promotes membrane fluidization and tight junction opening	Mild surfactant, well-tolerated	Limited availability, mostly used in pharmaceutical applications	(196, 219)
Choline Geranate (CAGE)	Paracellular (tight junction modulation, enzymatic protection, mucus viscosity reduction)	Strong bioavailability enhancer	Cost and regulatory concerns in some regions	(220, 221)

* Commercial products using these technologies with the objective of increasing absorption/bioavailability can be found in the market.

### 7.1 Phytase

**Figure 5:**
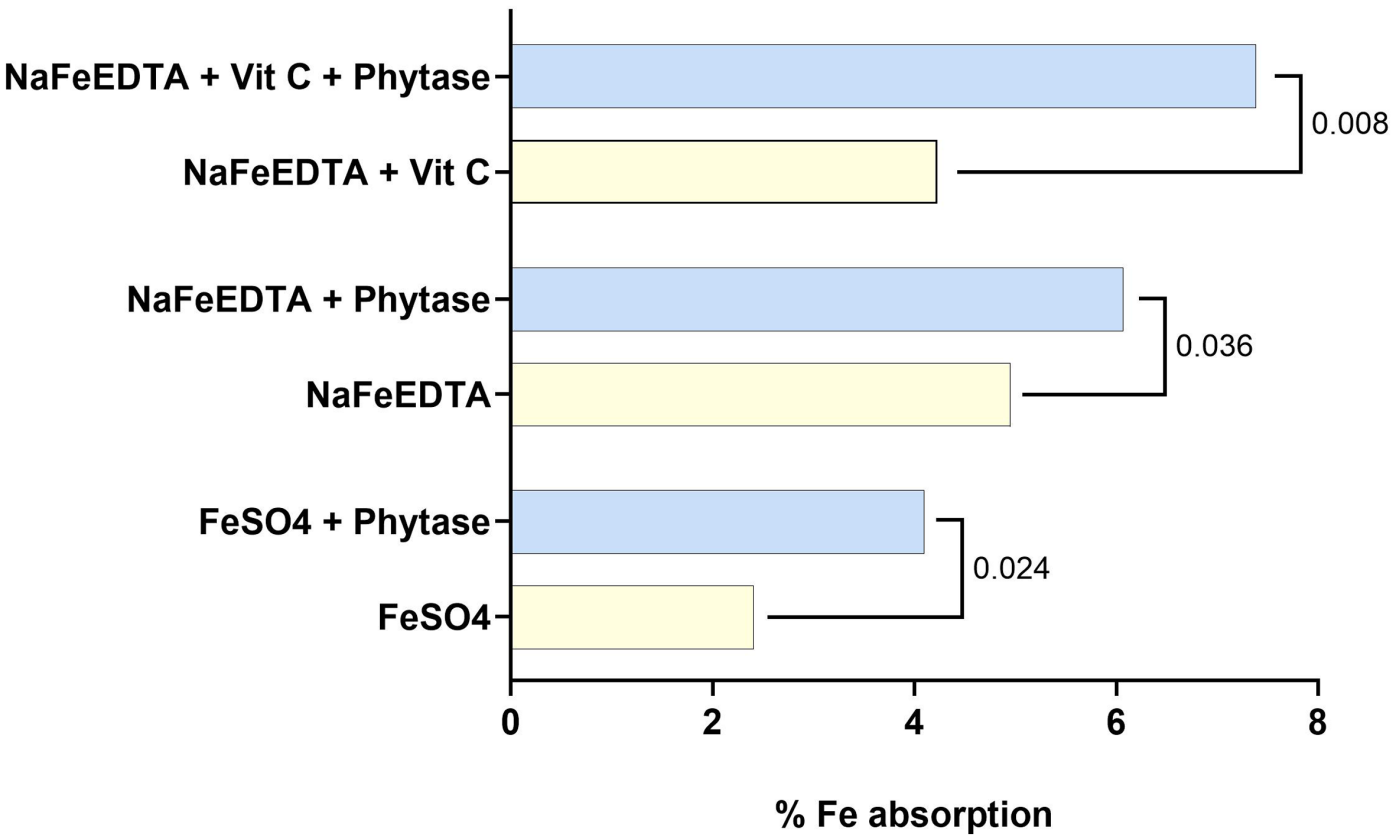
Bioavailability of iron is increased by phytase addition in diets containing iron from FeSO4, NaFeEDTA, or NaFeEDTA + vitamin C. Healthy young women were provided study diets (maize porridge) containing 3 mg stable labeled Fe as FeSO4, NaFeEDTA, or NaFeEDTA where 60 mg vitamin C was also added. Each diet was provided both with and without 10 mg phytase. Each woman consumed a diet with a single given iron source with or without phytase in a crossover design. Shown is the fractional iron absorption from each of the test diets, with the p value for each pairwise comparison. In each pairwise comparison, the presence of phytase significantly increased iron absorption. Figure was created using data from Troesch et al. (187).

Phytic acid is one of the most common inhibitors of mineral bioavailability, particularly in plant-based diets (43–45). Microbial phytase active at gut pH can break down phytates to a level where binding to minerals is significantly decreased (208, 209). Vitamin C is also a well-known enhancer of iron absorption (186). Many studies have shown that the presence of phytase when iron is present significantly increases iron absorption (43, 44, 187). In one study, the potential effect of phytase on iron bioavailability was studied when the diet contained iron as ferrous sulfate, as NaFeEDTA, or as NaFeEDTA with vitamin C also added. Phytase addition significantly increased the bioavailability of iron in all three diets (*p* < 0.05 for the diets containing ferrous sulfate or NaFeEDTA; *p* < 0.01 for the diet containing NaFeEDTA + vitamin C). The combination of phytase, vitamin C, and NaFeEDTA resulted in an iron absorption of 7.4%, compared with an absorption of 2.4% from FeSO4 without phytase or vitamin C in the same meal (*p* < 0.001) (Figure 5) (187). These findings highlight the potential of incorporating phytase into food fortification strategies, especially in plant-based diets where phytic acid is prevalent. By reducing mineral-binding phytates, phytase can enhance the bioavailability of key micronutrients like iron and zinc, making fortification efforts more effective in addressing micronutrient deficiencies. Indeed, phytase is expected to be introduced in flours, porridges, and other staple foods as a processing aid to increase iron bioavailability in certain countries in Asia and Africa. Furthermore, phytase has recently been launched in dietary supplements in several markets in Europe, the United States, and Asia including India and Taiwan (188).

### 7.2 Permeation enhancers

**Figure 6:**
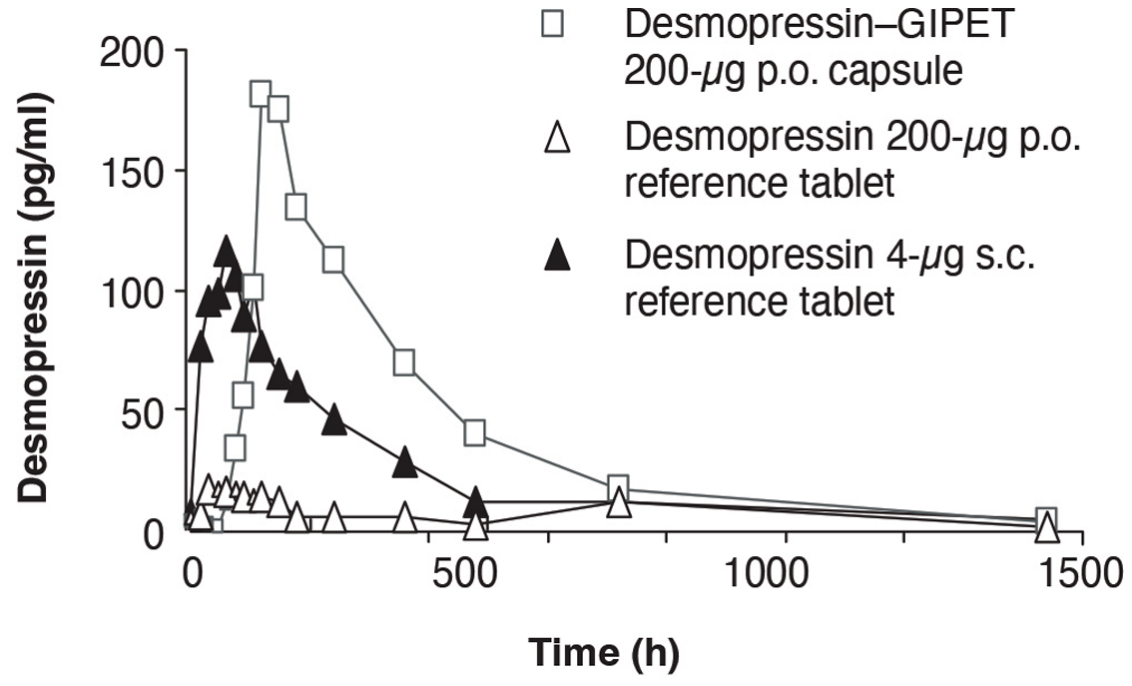
Oral bioavailability of a peptide with permeation enhancer technology. Plasma concentration–time profile of a peptide (desmopressin) following a single oral dose of 200 μg formulated with a permeation enhancer (GIPET™) compared to a 200 μg oral reference tablet without permeation enhancer, and a 4 μg subcutaneous injection. Data show significantly enhanced absorption with the permeation enhancer vs. the oral dose without, including a markedly higher peak concentration and prolonged systemic exposure. AUC analysis demonstrates a 12-fold increase in bioavailability (2.4% vs. 0.2%), supporting the effectiveness of medium-chain fatty acid–based permeation enhancement in improving oral peptide delivery. Reprinted by permission of Informa UK Limited, trading as Taylor & Francis Group, www.tandfonline.com, from Leonard et al., Promoting absorption of drugs in humans using medium-chain fatty acid-based solid dosage forms: GIPET™. Expert Opinion on Drug Delivery (195). GIPET™: gastrointestinal permeation enhancement technology (Merrion Pharmaceuticals, LLC); AUC, area under the curve; s.c., subcutaneous.

Several permeation enhancer (PE) technologies have been developed to increase absorption of nutrients and other compounds. PEs transiently alter the intestinal epithelial barrier to facilitate uptake of nutrients that normally would have low bioavailability. Nutrient absorption facilitated by PEs can be paracellular, transcellular, or both (189, 190). PEs can be derived from plants. Piperine, for example, is isolated from black pepper and can be found in many dietary supplements in the U.S. (191). Other PEs can be a combination of fatty acids or fatty acid derivatives (189, 190, 192). In one study looking at vitamin B_12_ pharmacokinetics, an N-acetylated amino acid derivative of salicylic acid (sodium N-[8-(2-hydroxybenzoyl)amino]caprylate, or SNAC) increased the rate of B_12_ uptake, and also increased the total uptake by over two fold (192). The SNAC technology is used in a medical food for the dietary management of patients with diagnosed vitamin B_12_ deficiency (193). Another example is the gastrointestinal permeation enhancement technology that uses medium-chain fatty acids like sodium caprate (C10) and sodium caprylate (C8) (189), reported to act via increasing paracellular uptake of molecules and potentially other mechanisms (189, 190, 194–196, 210). The beneficial effects of C10 and C8 on uptake have been described in the pharmaceutical space (Figure 6) (195), and this technology could be applied in dietary supplements as well.

### 7.3 Lipid-based formulations

**Figure 7:**
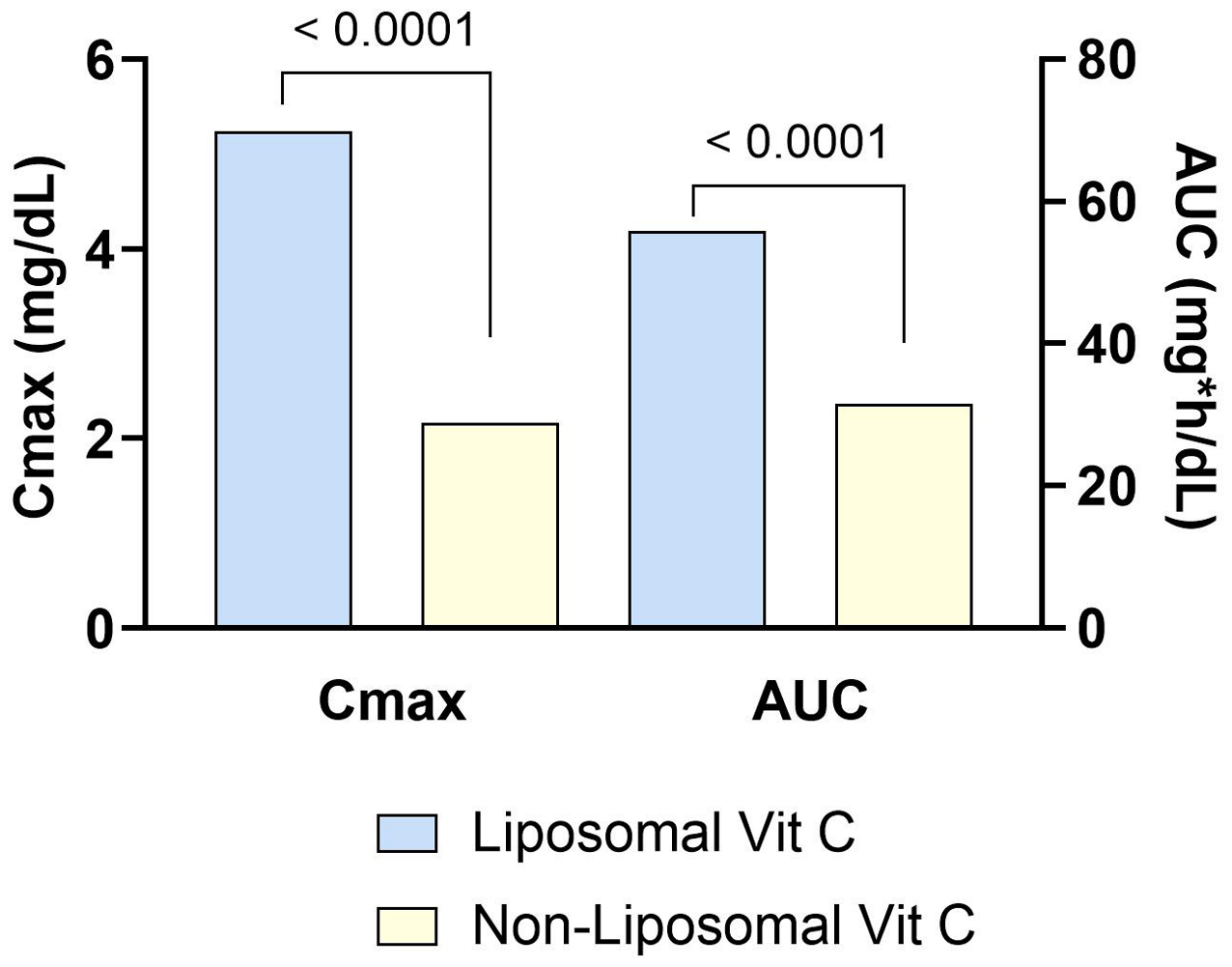
Liposome technology increases the bioavailability of vitamin C. Healthy adult subjects were provided with a single dose of liposomal or non-liposomal vitamin C (1,000 mg) in a crossover design. Blood was collected prior to vitamin C administration, and at 20 timepoints through 24 h post administration. Vitamin C concentrations were measured in the plasma. The maximum concentration of plasma vitamin C (Cmax) and AUC from 0 to 24 h are shown in the graph. p values for the comparisons between liposomal and non-liposomal sources are shown above the bars. Figure was created using data from Gopi and Balakrishnan (60).

Liposomes and other lipid-based formulations (LF) are other compelling technologies to increase nutrient bioavailability (60, 197, 198). Nutrients such as vitamins are loaded into matrices of phospholipids, which are the same types of lipids that form cell membranes. These structures protect the encapsulated nutrients from enzymes and gastric acid in the upper GI tract. In the small intestine the lipid matrix is exposed to bile salts and pancreatic lipase, forming micelles, allowing for the nutrient “cargo” to be released slowly from the matrix. This sustained release increases the allowable time for uptake and helps prevent the saturation of nutrient receptors in the small intestine, thereby increasing the proportion of nutrients that can be absorbed relative to nutrients that are not encapsulated. These technologies have been demonstrated to enhance the bioavailability of vitamin C, for example (Figure 7) (60, 197, 198). Commercial examples of products using lipid-based formulations can be found in pharma and dietary supplements.

### 7.4 Compounding/encapsulation

Finally, formulating or “compounding” micronutrients can have a substantial impact on bioavailability. Vitamins—especially the fat-soluble ones—can be encapsulated into “beadlets” or spray-dried into powders (199). Each process initially generates droplets of 1 micron or smaller, making them smaller than micelles, thus enhancing their absorption several times. The encapsulation also serves to protect the vitamins from oxidation and undesirable nutrient-nutrient interactions. Each process contains excipients such as gelatin, dextrin, sugar, and water, with the beadlets also bearing an outer starch shell via powder catch in a starch fluidized bed. A good example for this higher bioavailability is provided by the ratio of conversion of beta-carotene to Vitamin A. The US IOM (now NAM) reports that 12 micrograms of dietary beta-carotene are required to produce 1 μg of retinol (46). Yet, a form of pure beta-carotene in oil can achieve a conversion rate of 2.4:1, and a 1% water-dispersible beadlet can achieve a conversion ratio of 2:1 (internal dsm-firmenich data) (200).

These “enhanced forms” of highly bioavailable fat-soluble vitamin forms are present in many commercially available supplements and fortified staple foods that contribute to micronutrient adequacy. Twelve countries in the world have mandatory legislation for the fortification of sugar with vitamin A (201). This is done with a water-dispersible encapsulated form of vitamin A that—as explained above—is highly bioavailable (202). Chile has recently mandated the fortification of milk and wheat flour with vitamin D. The latter will use the same sort of powder vitamin forms with superior bioavailability (203, 204).

## 8 Conclusion

Micronutrient deficiencies remain a global public health challenge. Optimal intake and status of vitamins and minerals ideally would be accomplished via the consumption of diverse and well-balanced diets, but this has proven difficult to achieve. Indeed, billions of people have inadequate intakes for one or more of a broad array of micronutrients and deficiencies are widespread. Poor status contributes to a wide spectrum of adverse health outcomes.

Bioavailability—the proportion of a nutrient that is absorbed and can be used in metabolism or stored by the body for future utilization—is a critical determinant of nutritional status. For micronutrients, it is influenced by host factors such as life stage, nutritional status, physiological state, disease state, and the gastrointestinal microbiota; and dietary factors such as vitamin dose and form, nutrient interactions, and the dietary matrix including the presence of antagonists. These elements can conspire to limit the absorbability and thus the utilization of these nutrients and consequently are important contributors to the marked state of micronutrient deficiencies seen worldwide.

To address these gaps, proven strategies such as dietary supplementation and food fortification have significantly improved micronutrient intake and status across populations. In parallel, advancements in bioavailability-enhancing technologies—such as optimized nutrient forms (e.g., calcifediol, methylfolate), enzymes like phytase, permeation enhancers, liposomes/lipid based formulations, and compounding/encapsulation—offer powerful tools to further improve nutrient absorption and utilization. Looking ahead, innovations such as microbiota modulation and crop biofortification may provide additional avenues to support global nutritional sufficiency. Ensuring access to highly bioavailable nutrients is essential to closing nutritional gaps and advancing health equity, in alignment with the United Nations Sustainable Development Goals 2 and 3.
