# Supplementary material for: Micronutrient bioavailability: concepts, influencing factors, and strategies for improvement
Source: Front Nutr. 2025 Nov 19;12:1646750. doi: 10.3389/fnut.2025.1646750 (PMC12673670; doi:10.3389/fnut.2025.1646750)
Supplement: Supplementary file 1 [file Table_1.docx]

| **Supplementary Table 1.** Bioavailability of key micronutrients under different conditions: Dietary factors | | | | |
| --- | --- | --- | --- | --- |
| **Nutrient** | **Enhancing factor and food source** | **Inhibiting factor and food source** | **Impact and Mechanism** | **References** |
| **Water Soluble Vitamins** | | | | |
| Biotin |  | Avidin (Egg whites, raw eggs) | Not absorbed: Binds biotin, making it biologically unavailable | (11,25) |
|  |  | Food Matrix (Cereals) | Lower bioavailability: Although most dietary biotin appears to be protein bound in both meats and cereals, biotin in cereals appears to be less bioavailable | (10,25) |
|  |  | Biotinidase deficiency (Protein-bound biotin foods) | Reduces absorption: Lack of adequate digestion of protein-bound biotin | (25) |
| Folate |  | Chemical Form (Polyglutamates in fresh food) | Lower absorption: Synthetic monoglutamate form (i.e., folic acid) used as fortificant and supplements is better absorbed | (11) |
|  |  | Chemical Form (Food folate vs. folic acid) | Lower absorption: Food folate bioavailability of approx. half that of folic acid taken on empty stomach or folic acid in fortified food | (25) |
|  |  | Fiber (Wheat bran) | Reduces absorption: Certain forms of fiber may decrease the bioavailability of certain forms of folate under some conditions | (25) |
|  | Chemical Form, MTHF (Supplements and fortified foods |  | Higher bioavailability: At 400 μg/day, 5-MTHF was found to be more bioavailable than folic acid and a conversion factor of 2 is proposed for this intake level and for higher intakes | (222) |
| Niacin |  | Chemical Form (Niacin in mature corn) | Not absorbed: Unavailable for absorption | (11) |
|  |  | Food Matrix (Animal vs. plant-based) | Affects bioavailability: The bioavailability of niacin in animal-sourced foods (67%) was generally greater than that in plant-based foods (57%). | (10) |
|  |  | Food Matrix (Mature cereal grains) | Reduces absorption: Largely bound and thus is only about 30 percent available | (25) |
| Pantothenic Acid |  | Chemical Form in food (Typical mixed diet) | Reduces absorption: Values of 40 to 61 percent (mean 50 percent) have been given for absorbed food-bound pantothenic acid, compared to the crystalline vitamin | (25) |
|  | Food Matrix (Animal -based vs plant-based) |  | Affects bioavailability: Pantothenic acid is more bioavailable in animal-sourced foods (80%) than in plant-based foods (50%) | (10) |
| Riboflavin | Food intake (All foods) |  | Increases absorption: The rate of absorption is proportional to intake, and it increases when riboflavin is ingested along with other foods and in the presence of bile salts | (25) |
| Thiamin |  | Polyphenols (Certain cereals [red sorghum], legumes [red kidney beans, black beans, black grams], spinach, betel leaves, oregano; Beverages: tea, coffee, cocoa, red wine) | Reduces absorption: Some polyphenols inactivate thiamin | (11) |
|  |  | Thiaminases (Fish, shellfish, Brussels sprouts, red cabbage) | Destroyed: Destroys thiamin | (11) |
| Vitamin B_12_ | Food Matrix (Different foods) | Food Matrix (Different foods) | Affects bioavailability: Different levels of absorption are assumed under various conditions | (25) |
|  | Food vs. synthetic (Animal protein) |  | Favor synthetic: Higher bioavailability of synthetic B_12_ than of protein-bound B_12_ for a substantial proportion of older adults. | (25) |
|  |  | High Intake (All animal foods) | Lower bioavailability: Intrinsic factor-mediated intestinal absorption is estimated to be saturated at about 1.5–2.0 µg per meal under physiological conditions, leading to a decrease in B_12_ bioavailability with increasing intake. | (223) |
|  | Food source (Type of animal food source) |  | Affects bioavailability: The bioavailability of B_12_ varies among food sources: 30–42% for fish meat, 52–89% for sheep meat, 61–66% for chicken meat, less than 9% to 36% for eggs, and 4.5–49% for liver products. Milk has a bioavailability of 65%. | (223) |
|  |  | Inactive analogs (Shellfish like abalones and whelks) | No vitamin activity: For individuals whose dietary habits primarily rely on consuming foods with inactive B_12_ analogs or those with low bioavailability of vitamin B_12_, they are at risk of not absorbing enough B_12_ | (223) |
| Vitamin B_6_ |  | Chemical Form in food (Typical mixed diet) | Affects bioavailability: Pyridoxine glucoside, is about 50% as bioavailable as the other B_6_ vitamins | (25) |
|  | Food Matrix (Animal-based vs plant-based) |  | Higer bioavailability in animal food: The bioavailability of vitamin B_6_ from animal products is quite high, reaching 100% for many foods. In general, the bioavailability from plant foods is lower. The presence of fiber reduces the bioavailability by 5–10% | (11,224) |
|  |  | Pyridoxine glucoside (Cruciferous foods and others) | Decreases bioavailability: The presence of pyridoxine glucoside reduces the bioavailability by 75–80% | (224) |
| Vitamin C |  | High doses (Supplements) | Reduces absorption: Bioavailability diminishes at higher doses, and is less than 50% at doses exceeding around 1,000 mg | (225) |
| **Fat soluble vitamins** | | | | |
| Carotenoids | Fat (Foods containing α-carotene, β-carotene, lycopene, lutein, zeaxanthin, and β-cryptoxanthin) |  | Increases absorption: The presence of fat in the small intestine stimulates the secretion of bile acids from the gall bladder and improves the absorption of carotenoids by increasing the size and stability of micelles, thus allowing more carotenoids to be solubilized. | (225) |
|  | Cooking (Foods containing α-carotene, β-carotene, lycopene, lutein, zeaxanthin, and β-cryptoxanthin) |  | Increases absorption: More digestible cooked food releases carotenoids more readily | (225) |
| Fat soluble vitamins |  | Dietary Fiber (Unrefined cereals, legumes, nuts, oilseeds, fruits, and vegetables) | Reduces absorption: Lignin and pectin bind bile acids | (11) |
|  | Fat (Fats and oils, animal adipose tissue, milk and milk products, vegetables, seeds, nuts) |  | Increases absorption: Products of fat digestion (fatty acids, monoglycerides, cholesterol, and phospholipids) plus bile salts solubilize fat soluble vitamins and carotenoids in intestinal milieu | (11) |
| Pro-Vitamin A | Lutein (Broccoli, eggs, paprika, supplements) |  | Increases absorption: Lutein given in combination with β-carotene significantly increased β-carotene serum concentrations compared to when β-carotene was given alone | (46) |
| Vitamin A | Food Matrix (Different factors in different foods) | Food Matrix (Different factors in different foods) | Affects bioavailability: Absorption and bioconversion of ingested provitamin A carotenoids to retinol:12:1 for dietary all-trans-β-carotenes, 2:1 for supplemental all-trans-β- carotene, 24:1 for other dietary provitamin A carotenoids | (46) |
|  | Fat (Oily and fatty foods) |  | Increases absorption: Dietary vitamin A is digested in mixed micelles and absorbed with fat, which has been shown to improve retinol and carotene absorption | (46) |
|  |  | Iron (Iron poor diets) | Decreases bioavailability: Iron deficiency possibly alters the distribution of vitamin A concentration between plasma and liver | (46) |
|  |  | Zinc (Zinc poor diets) | Decreases bioavailability: Zinc deficiency influences the mobilization of vitamin A from the liver and its transport into the circulation | (46) |
|  |  | Alcohol (Alcoholic beverages) | Decreases stores: Ethanol consumption results in a depletion of hepatic vitamin A concentrations in animals and in humans | (46) |
| β-carotene | Food Matrix (Supplements vs Dark green leafy vegetables, yellow and orange vegetables) |  | Affects bioavailability: The absorption of β-carotene from supplements is significantly higher than from carrots, broccoli, or green leafy vegetables | (46) |
|  | Food Matrix (Cooked vs Raw: Dark green leafy vegetables, yellow and orange vegetables) |  | Affects bioavailability: Absorption of carotene is higher from homogenized carrots than sliced carrots, and higher from cooked vs. raw carrots or spinach | (46) |
| Vitamin D | Chemical Form (Animal (D_3_) vs. vegan (D_2_) forms |  | Higher bioavailability in D_3_: Vitamin D_3_ elevates serum levels faster and maintains them for a longer period, at high doses | (32) |
|  | Chemical Form (Calcifediol vs. D_3_) |  | Higher bioavailability of Calcifediol: Calcifediol is estimated to be 2.5-3 times more effective than cholecalciferol at raising circulating 25(OH)D levels | (56,154) |
|  | Fat (Oily and fatty foods) |  | Increases absorption: The efficient absorption of vitamin D is dependent upon the presence of fat in the lumen, which triggers the release of bile acids and pancreatic lipase | (32) |
| Vitamin E |  | Non-alpha forms (Oily foods in general) | No vitamin E activity: Of the eight naturally occurring forms of vitamin E only the α-tocopherol form of the vitamin is maintained in human plasma | (225) |
|  |  | Racemic 2R, 2S mixtures (Synthetic vitamin E) | 50% vitamin E activity: Vitamin E is defined and limited to the 2R-stereoisomeric forms of α-tocopherol | (225) |
|  | Fat (Foods high in fats and supplements) |  | Increases absorption: Vitamin E absorption requires micelle formation and chylomicron secretion by the intestine | (225) |
|  | Vitamin C (Diet and supplements) |  | Boost antioxidant function: Tocopheroxyl radical is formed in antioxidant function, and this radical can be reduced by ascorbic acid, returning vitamin E to its reduced state | (225) |
|  | Polyunsaturated fat (PUFA rich foods) |  | Increases requirement: It was suggested that a ratio of at least 0.4 mg (1 µmol) α-tocopherol per gram of PUFA should be consumed by adults | (225) |
| Vitamin K | Fat (Oily and fatty foods) |  | Increases absorption: Vitamin K in the diet, is absorbed in the jejunum and ileum in a process that is dependent on the normal flow of bile and pancreatic juice and is enhanced by dietary fat | (46) |
|  | Food Matrix (Free vitamin K vs. food) |  | Favors free Vitamin K: Studies suggest that vitamin K in food sources is less well absorbed. Phylloquinone in the form of cooked spinach was reported to be 4 percent as bioavailable as that from a phylloquinone supplement | (46) |
|  | Vitamin Form (Vitamin K containing foods) |  | Affects bioavailability: Both K_1_ and MK-7 (form of K_2_) are absorbed well, with longer half-life and greater accumulation during prolonged intake for MK-7. MK-7 has been reported to be more effective than K_1_ at carboxylating osteocalcin | (226) |
| **Minerals** | | | | |
| Ca, Fe, Zn | Chemical Form (Preformed peptide–mineral complexes) |  | Increases absorption: solubility, bioavailability, transport, depending on the mineral | (35) |
| Iron | Chemical Form, heme vs. non-heme (Meat, poultry, fish) |  | Increases absorption: Heme iron (bound in a porphyrin ring) in hemoglobin and myoglobin from meat, poultry, and fish is more readily absorbed than nonheme iron found in foods of plant and animal origin | (11) |
|  | Chemical Form (Ferrous) | Chemical Form (Ferric, Elemental, ferric phosphate) | Affects bioavailability: Freely water-soluble salts are more bioavailable than the poorly soluble ones. Water insoluble salts soluble in dilute acids are even less bioavailable. Elemental iron exhibits a bioavailability that is dependent on the production method | (36) |
|  | Chemical Form (Amino-Chelate supplements) |  | Improves bioavailability: Ferrous bisglycinate shows some benefit over other iron supplements in increasing hemoglobin concentration and reducing GI adverse events among pregnant women | (37) |
|  | Chemical Form (Iron Sodium EDTA) |  | Improves bioavailability: Iron from ferric sodium EDTA is 2 to 3 times more bioavailable than from other mineral sources. | (38) |
|  | Ascorbate (Dietary or supplement) |  | Increases absorption: Chelate with ferric iron at acid pH that remains soluble at the alkaline pH of the duodenum | (21) |
| Zinc | Food Matrix (Refined vs. unrefined |  | Affects absorption: Zinc bioavailability from a mixed or vegetarian diet based on refined cereal grains is estimated to be 26–34%, whereas 18–26% is absorbed from an unrefined cereal-based diet | (227) |
|  | Food Matrix (animal vs. plant-based diet) |  | Affects absorption: Human zinc absorption is substantially higher in the presence of protein from animal sources than plant-based protein and the addition of animal protein to vegetable-based food significantly improved its zinc bioavailability in vivo | (227) |
|  | Chemical Form (Bis-glycinate vs. gluconate) |  | Increases bioavailability: Bis-glycinate significantly increased the oral bioavailability of zinc (+43.4%) compared with the gluconate | (228) |
|  | Low intake level (Amount of absorbable zinc in diet) | High intake level (Amount of absorbable zinc in diet) | Affects balance of absorption and excretion: As absorbable zinc intake increases, absorption rate is decreased. Intestinal excretion also modulated | (21) |
| Zn, Fe, Ca, Mg |  | Phytate (Unrefined cereals, legumes, nuts, oil seeds) | Lowers absorption: Binds certain cations to form insoluble complexes in gut | (11) |
|  |  |  |  |  |
| **All micronutrients** | | | | |
| All | Supplements (Supplements generally better absorbed than dietary sources) |  | Micronutrients often bound to food matrix or antagonists | (10,21,162) |
| Vitamins, minerals, and choline | Food matrix (Animal vs. plant-based) |  | In general, a wide variety of vitamins and minerals are more available from animal than from plant sources: Increased binding to anti-nutritional factors in plants (phytate, fiber, etc.); lack of B_12_ in plant-based foods; Supplementation/fortified foods are recommended by expert groups including WHO | (10,49) |
